# Unique Signatures of Natural Background Radiation on Human Y Chromosomes from Kerala, India

**DOI:** 10.1371/journal.pone.0004541

**Published:** 2009-02-26

**Authors:** Sanjay Premi, Jyoti Srivastava, Sebastian Padinjarel Chandy, Sher Ali

**Affiliations:** Molecular Genetics Laboratory, National Institute of Immunology, Aruna Asaf Ali Marg, New Delhi, India; Buck Institute for Age Research, United States of America

## Abstract

**Background:**

The most frequently observed major consequences of ionizing radiation are chromosomal lesions and cancers, although the entire genome may be affected. Owing to its haploid status and absence of recombination, the human Y chromosome is an ideal candidate to be assessed for possible genetic alterations induced by ionizing radiation. We studied the human Y chromosome in 390 males from the South Indian state of Kerala, where the level of natural background radiation (NBR) is ten-fold higher than the worldwide average, and that from 790 unexposed males as control.

**Results:**

We observed random microdeletions in the Azoospermia factor (*AZF*) *a*, *b* and *c* regions in >90%, and tandem duplication and copy number polymorphism (CNP) of 11 different Y-linked genes in about 80% of males exposed to NBR. The autosomal homologues of Y-linked *CDY* genes largely remained unaffected. Multiple polymorphic copies of the Y-linked genes showing single Y-specific signals suggested their tandem duplication. Some exposed males showed unilocus duplication of *DAZ* genes resulting in six copies. Notably, in the *AZFa* region, approximately 25% of exposed males showed deletion of the *DBY* gene, whereas flanking genes USP9Y and *UTY* remained unaffected. All these alterations were detected in blood samples but not in the germline (sperm) samples.

**Conclusions:**

Exposure to high levels of NBR correlated with several interstitial polymorphisms of the human Y chromosome. CNPs and enhanced transcription of the *SRY* gene after duplication are envisaged to compensate for the loss of Y chromosome in some cells. The aforesaid changes, confined to peripheral blood lymphocytes, suggest a possible innate mechanism protecting the germline DNA from the NBR. Genome analysis of a larger population focusing on greater numbers of genes may provide new insights into the mechanisms and risks of the resultant genetic damages. The present work demonstrates unique signatures of NBR on human Y chromosomes from Kerala, India.

## Introduction

Natural background radiation (NBR) has been affecting all forms of life since the time of its inception, although its geographical scope has been varied. Semi-permanent exposure to ionizing radiation leaves a lasting imprint on the genome [1]–[3]. Such change(s) may be used as biomarkers to monitor progression of tumors, chromosomal lesions, minisatellite length polymorphisms, and other alterations involving DNA [4]. Attempts have been made to establish a correlation between background radiation and phenotypic changes in rats [5], cases of Down's syndrome [6], chromosomal aberrations [7], and congenital malformations [8]. However, experimental evidence for radiation-induced mutations/alterations in humans still remains controversial in the absence of sufficient data.

The human Y chromosome, with fewer than 50 genes or gene families coding for proteins, is not essential for life but harbors several testis-specific genes [9] necessary for sperm production, and hence continuation of the species. It is well known that at least three non-overlapping regions of the human Y chromosome – *AZFa*, *AZFb*, and *AZFc* (Azoospermia factors *a*, *b*, and *c*) – are essential for spermatogenesis [10]. Microdeletions in these regions affecting one or more of the candidate genes (*DAZ*, *RBMY*, *DBY*, and *USP9Y*) cause male infertility [11]. Deletions of the *DAZ* genes in the distal Yq11 (*AZFc*) region are always associated with azoospermia [12]–[14]. The human Y chromosome abnormalities have also been attributed to the single copy *SRY* gene located on the p11.3 region, which plays a predominant role in male sex determination [15]. Mutations in the conserved HMG box domain and its up/down stream sequences have been correlated with sex reversal or poor binding of the *SRY* protein to the target DNA [15].

Assessment of the effects of long-term experimental irradiation on humans and its impact across the generations is not possible owing to logistic and ethical constraints. Only a few studies have been carried out following the Chernobyl disaster focusing on the effect of radiation on minisatellite mutation rate [16] or the radiation effect from nuclear weapon tests on the human germline mutation rate [3]. In this context, coastal areas in Kerala (South India), which contain 10% thorium phosphate monazite, offer a natural setting to assess the radiogenomic effects of background radiation. This radioactivity strip measuring an area of about 10 km by 1 km supports a sizable population of fishermen [17]. Earlier, we reported on the status of *DAZ* genes in 100 males exposed to NBR [18]. Triggered by the initial results, we undertook analysis of the Y chromosome in 390 exposed males and 790 unexposed ones as controls for possible structural variations. The results showed exclusive somatic microdeletions and CNP of the Y chromosome–linked genes.

## Results

### Random Y Chromosome Microdeletions in NBR-Exposed Males

**Figure 1:**
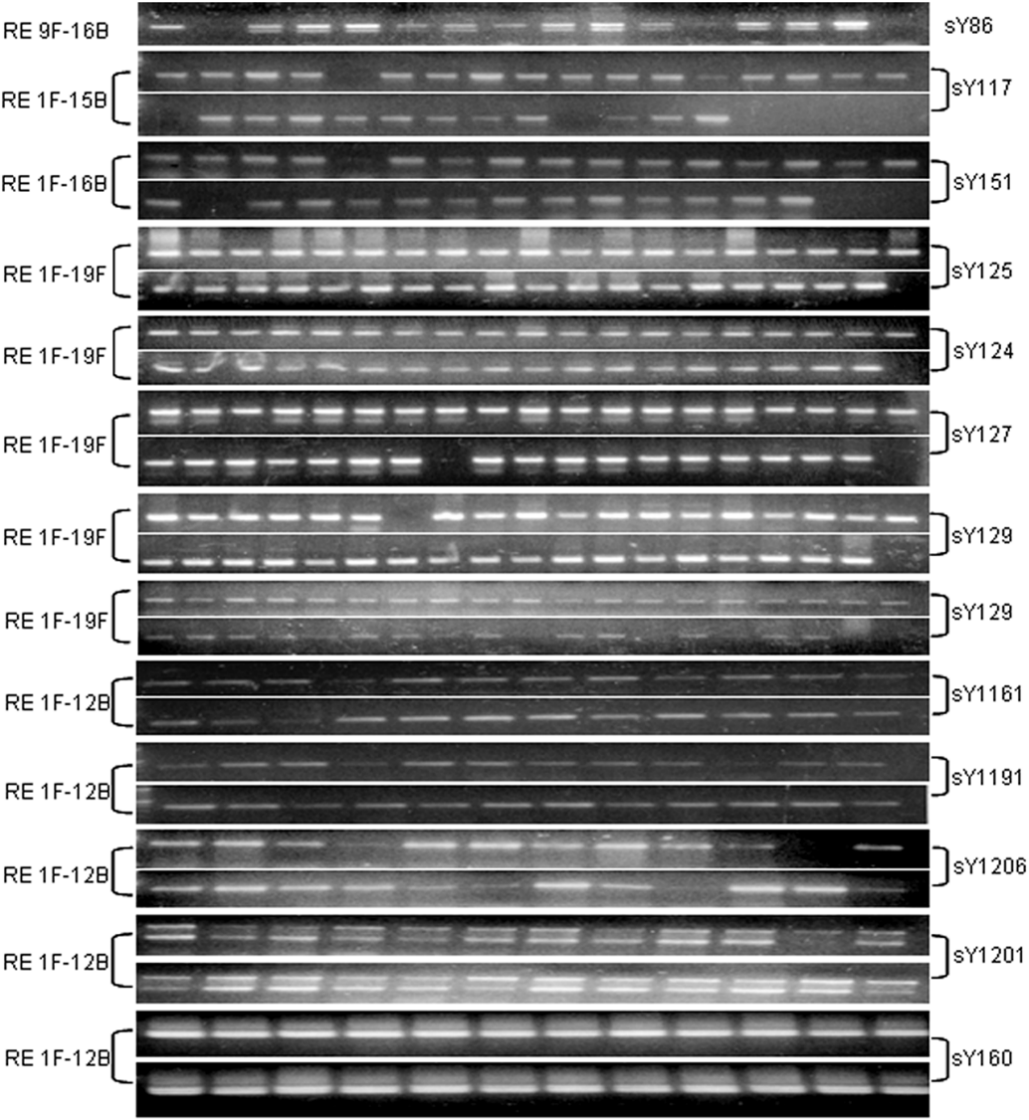
PCR-based assays of STSs with DNA of a few representative males exposed to natural background radiation (NBR). On the left of the panels, “RE” denotes radiation exposed, F and B denote father and son, respectively, and numbers refer to family IDs. The STSs are given on the right of the panels. Note *de novo* microdeletions in father but not in son, and vice-versa.

**Figure 2:**
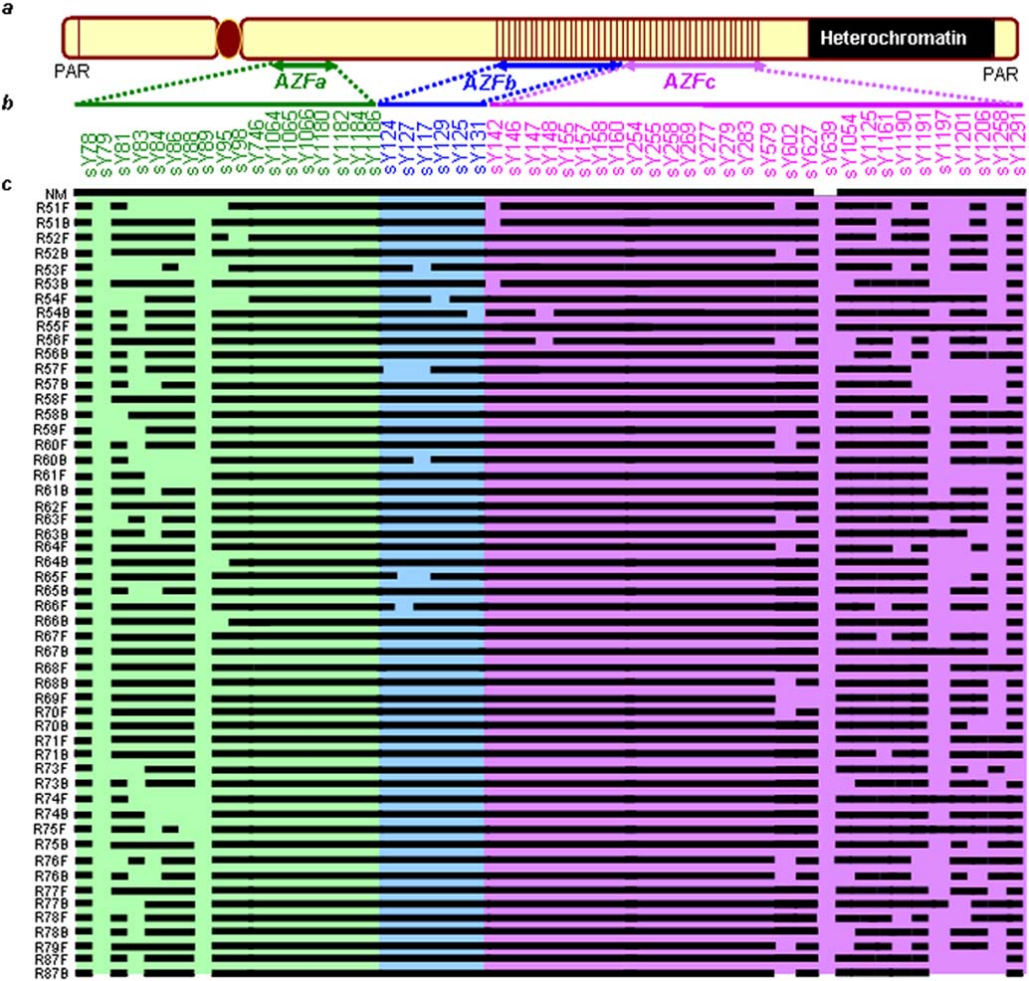
STS map for *AZFa*, *b* & *c* regions in representative exposed males. (a) Conventional organization of the human Y chromosome. (b) STSs employed in mapping three different *AZF* regions are shown in corresponding colors. (c) Results of STS mapping using genomic DNA from the peripheral blood lymphocytes of the exposed males. “NM” denotes unexposed males. Sample IDs for the exposed males are given on left. “F” and “B” denote father and son, respectively. Note deletion of sY79, sY89 and sY639 in all the males and frequent deletions of sY83 and sY84 in *AZFa*, sY117 and sY129 in *AZFb* and several STSs in distal and proximal *AZFc* regions (see text for details).

STS mapping showed randomly scattered microdeletions in the *AZFa*, *AZFb* and *AZFc* regions of the Y chromosome in the exposed males. Frequency of microdeletions was higher in the *AZFc* region than in *AZFa* and *AZFb*. The *AZFc* microdeletions were distributed mainly in the proximal and distal portions throughout the sample pool. These microdeletions included sY1197, sY1258, sY1206 and sY1201 STSs in about 90% of exposed males (Figures 1 and 2). None of the samples showed the characteristic STS profile of *gr/gr* (sY1291 negative; and sY1161, sY1206, sY1191 and sY1201 all positive) or *b1/b3* (sY1161, sY1197, sY1191 and sY1291 all negative; and sY142, sY1258, sY1206 and sY1201 all positive) deletions/duplications, except for a single sample (34B) depicting *gr/gr* deletion. In the absence of a blood sample from this male, FISH analysis could not be conducted. The *AZFa* microdeletions were confined to the proximal region encompassing sY79 and sY81 STSs (deletion interval 5A), sY88 (deletion interval 5D), and sY86 in about 85% of exposed males. Absence of sY117 in some exposed males hints at the *AZFb* phenotype, but the presence of sY149, sY127, and other interstitial STSs proved the intactness of the *AZFb* region. STS mapping of *AZFa*, *b* and *c* regions in representative exposed males is given in Figure 2.

**Figure 3:**
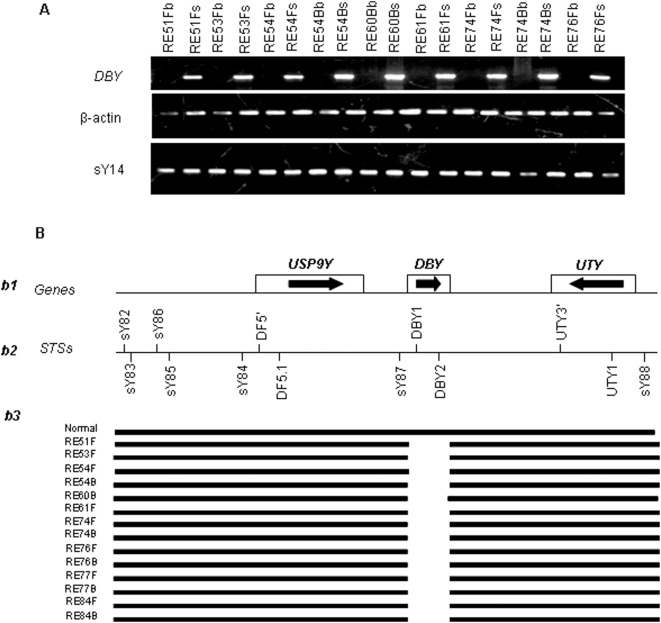
Exclusive somatic deletion of *DBY* gene in ∼25% of exposed males. (A) Representative males with their IDs shown on top. DNA from blood and semen samples are denoted as “b” and “s”, respectively. Note deletion of *DBY* amplicons exclusively in the blood DNA of exposed males compared to that of semen DNA. The *SRY* (sY14) and β-actin PCRs were used as positive controls. (B) Schematic representation of the *AZFa* region. Candidate *AZFa* genes are given in “*b1*”, STSs used in “*b2*”, and the results of screening in “*b3*”. Note the absence of amplicons with primers corresponding to *DBY1* and *DBY2*.

**Figure 4:**
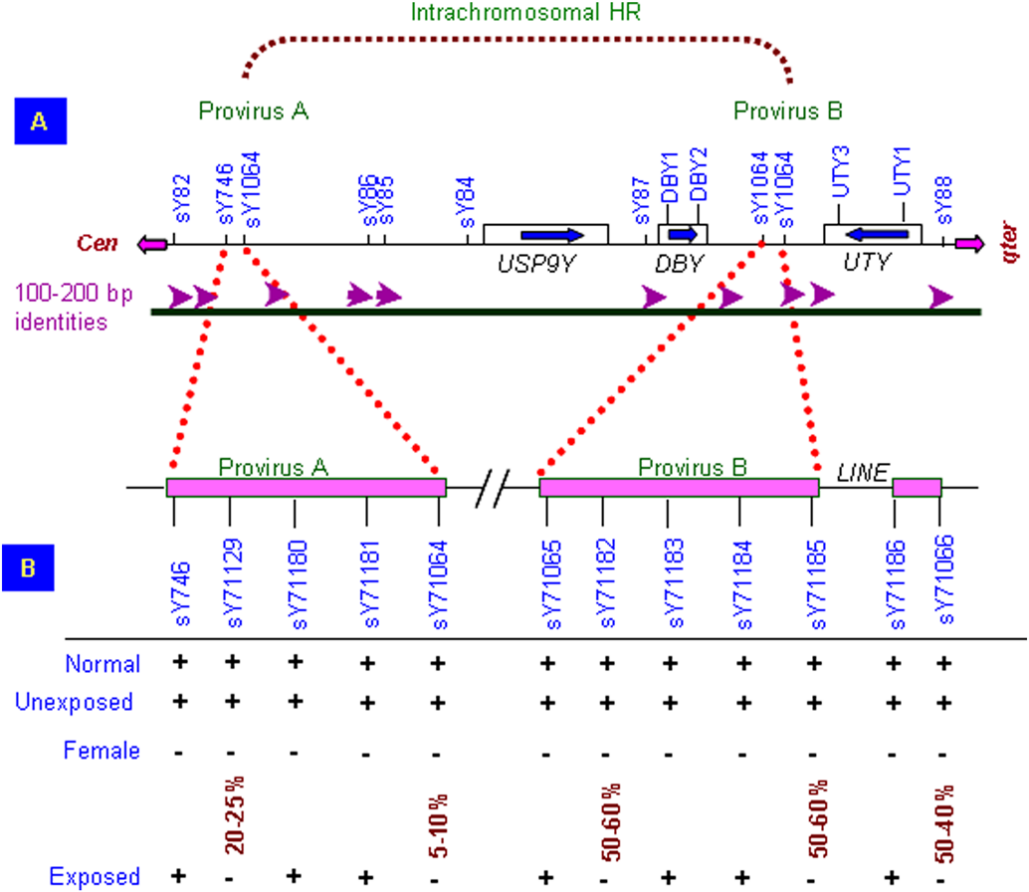
Analysis of the *AZFa* HERV elements in the exposed males. (A) Schematic representation of provirus elements with various STS markers used to study recombination mediated duplication or deletion. (B) Status of HERV elements in exposed males. Note presence of all the STSs in normal/unexposed males and randomly scattered microdeletions without any conclusive major deletion/duplication in the exposed ones. Female DNA was used as negative controls. Number of males showing deletion of a particular STS is given in percentage.

Besides random microdeletions, primers specific to *DBY1* and *DBY2* genes did not show any amplification in 95 exposed males (Figure 3). This was confirmed by Southern blot hybridization using ^32^P-labeled PCR product of *DBY* gene(s) from the normal male (not shown). Successful PCR amplification with sY83, sY86, sY84, DF1.5, sY87, *UTY1*, *UTY3* and sY88 STSs showed breakpoints located downstream of sY87 and upstream of *UTY3*. The *DBY* microdeletions were inconsistent between fathers and sons where father lacked them but son did not or *vice versa*. Studies on Human Endogenous Retroviral (HERV) elements also showed randomly scattered microdeletions without a conclusive provirus A/B mediated recombination (Figure 4). The STSs sY1066, sY1182 and sY1185 that were absent in approximately 50–60% of exposed males represented hotspots for microdeletions. Approximately 10% of *AZFc* and 4–5% of *AZFa*/*b* STSs showed additional amplicons along with the expected ones (Figure S1) involving sY1201, sY1206, sY83 and sY84 STSs in more than 98% of exposed males. Such amplicons were absent in the normal males and, as expected, in the females, suggesting creation of new primer binding sites in the exposed males.

The intactness of 95% of STSs in germline (sperm) DNA in the exposed males substantiated the somatic nature of microdeletions, corroborating our earlier study [18]. To rule out possibilities of PCR reaction failure, single and multiplex PCRs were conducted thrice followed by Southern blot hybridization using [^32^Pα-dCTP] labeled PCR products of the corresponding STSs of the unexposed males (not shown). Except for 2% of cases of the normal males that showed random microdeletions, all the unexposed control males (390 from Kerala and 400 from other parts of India) were free from microdeletions.

### Copy Number Polymorphism (CNP) of the Y-Linked Genes in Exposed Males

Real time PCR using TaqMan/SYBR green chemistries uncovered duplications/multiplications of the Y-linked genes resulting in CNPs. Similar CNPs of the *SRY* and *DAZ* genes were reported earlier [18], [19]. In the present study, TaqMan assays were standardized for 11 genes listed in Table 1. A ten-fold dilution series of genomic DNA showed a difference of 3.32–3.6 Ct (cycle threshold) per dilution and a standard curve with a slope of −3.24, both of which reflected maximum efficiency of the assay system (Figure S2). Representative real time PCR amplification plots for few assays are shown here (Figures S3, S4, and S5). As expected, no amplification was detected with female genomic DNA, confirming Y chromosome specificity of the primers and probes. All the samples were used in triplicate and each showed matching Ct values with a ±0.05 difference. Owing to the haploid status of the Y chromosome, Ct value for the *RNase*P gene was found to be −1 as compared to that of the *SRY* gene (ΔCt = Ct *SRY*-Ct *RNase*P = 1) and +1 for *DAZ* genes (ΔCt = −1) in blood DNA of the normal male [18], [19].

**Table 1:** Details of the primers and probes used in this study#.

Primers and TaqMan Probes for Copy Number Assessment of the Respective Genes				
Name	Entrez ID	Location	Size (bp)	Primers and Probes (5′-3′)
*CDY1*	9085	Yq11.23	3991	F (3626–4646) GTGGATGATGGCACCTTTGTG
				R (3676–3700) GCAGCCTGTAAGATGGGTTTGTAAA
				P (3656–3672) CTTGAGCCTGCTTTTC
*CDYL*	9425	6p21.5	250637	F (555–575) CCCTGACTGATGAGCAAACCA
				R (609–632) TCACTGAACTCCATGTGTGTTACC
				P (592–606) CCACTGGGCCTCTCG
*CDYL2*	124359	16q23.2	201738	F (102192–102216) GGTTTGCAAATAATGCCCCTGAAAA
				R (120272–120293) AGTGCCTCTCATCCTTCTCAGA
				P (120236–120250) CCGGCGTCCCCATTT
*UTY*	7404	Yq11	233495	F (1771–1787) CCATCACCCGCCTGGTT
				R (1821–1839) TCATCAACGTGGGCAAGCT
				P (1795–1811) CCTTCCCGGAGAGTATC
*VCY*	9084, 652821	Yq11.221	1942	F (1231–1253) CCTATCTCCCTGAGCAGCAACTA
				R (1277–1297) CCCTGCTGGTGAGATCTCTGA
				P (1254–1269) CAGCTGGGCCTAAACT
*BPY*	442868	Yq11.223	22403	F (21336–21356) TGGAGTCTGCCAAAACAAGGG
				R (21447–21468) CAGAGCAGGAGAGTCTCATCAC
				P (21362–21388) CACATATTGCGGAGTCCAGCACCCAGG
*HSFY*	442479	Yq11.222	4234	F (1060–1084) TCAATGAGGCTCCTTATCCTAACCT
				R (1117–1141) GCAGCCGATGTATCAAATGTCATAG
				P (1089–1103) CCAGCAGGCAACCAG
*SRY*	6736	Yq11.3	2045	Commercial assay from ABI (part number Hs00243216_s1)
*DAZ*	1617, 57135,	sY587 (STS)		F (220–249) TGACTGGACACCTAGTTTCATGAAC
		Yq11.223		R (299–320) GTCAAGAGGCATCAAGTGAAAGTTG
				P (256–279) CACCCTGTCTCCAACCC
*PRY*	9084	Yq11.223	25452	F (807–827) CAGGATGAAGGGATGCAGTGA
				R (847–869) CTTAGAGGTGGGTGTCAGTGAAA
				P (831–846) CAAGAGCCCAACCTTC

Primers Used for Cloning and Sequencing			
Gene	Primers	Tm	Amplicon
*SRY*	SRY1F- 5′GACAATGCAATCATATGCTTCTGC3′	65	600
	SRY1R- 5′CTGTAGCGGTCCCGTTGCTGCGGT3′		
	SRY3F- 5′GAATCTGGTAGAAGTGAGTTTTGGA3′	63	828
	SRY3R- 5′ TAAGGCCTTTATTAGCCAGAGAAAA3′		
	SRY4F- 5′CTTCTGCTATGTTAAGCGTATTCAA3′	63	599
	SRY4R- 5′CAGCTTTGTCCAGTGGCTGTAG3′		
*CDY1*	CDY1F-5′AAAGCTTTCTGTACTACACCAGAGGGTTG3′	68	2763
	CDY1R-5′AAGAAGTTTCTGCCTTTAATAATGTGTCCA3′		
*CDY2*	CDY2F-5′AAAGCTTTCTGTACTACACCAGAGGGTTG3′	*68.0*	*1954*
	CDY2R-5′AGCAGACAGACTGACAATTAAAACTCATCA3′		
*VCY*	VCY1F-5′TAGTGGAGTGTTGACCAATCACAG3′	*65*	*960*
	VCY1R-5′ACACACCACCTCTTCCTTCCTC3′		
*HSFY*	HSFYF-5′ATGCAGGCTGGAAGAGTAGCTAAAGAAATA3′	*68*	*2100*
	HSFYR-5′TGGTGGAAGATATGTTGTAATTAGGGTGAT3′		

# F and R refer to forward and reverse primers; P is the TaqMan probe.

TaqMan probes uncovered CNPs in most of the Y-linked genes (*SRY*, *DAZ*, *VCY*, *CDY1*, *UTY*, *HSFY*, *PRY*, *XKRY*, *BPY2*) and DYZ1-repeat showing a maximum of 2–3 rounds of duplication in ∼85% of exposed males (Table 2). Most frequently duplicated genes included *DAZ*, *CDY1* and *PRY*. However, copies of autosomal (*CDYL1*, *CDYL2*) genes largely remained unaffected. The autosomal genes *CDYL1* and *CDYL2* showed normal copies in about 95% of cases, whereas those of retro-transposed homolog *CDY1* was found to be frequently altered. Often, there was no correlation between father and son (denoted as f = father, b = son) with respect to the number of copies of this gene. The germline DNA of exposed males showed normal copies of these genes in >90% of males. In all the cases, copy number never decreased; it either increased or remained unaltered.

**Table 2:** Copy number polymorphism of the Y-linked and autosomal genes in human males exposed to natural background radiation (NBR)#.

Y-linked												Autosomal	
Sample ID	*SRY*	*DAZ*	*CDY1*	*PRY*	*UTY*	*HSFY*	*VCY*	*BPY2*	*XKRY*	*TSPY*	*DYZ1*	*CDYL*	*CDYL2*
**Normal males**	**1**	**4**	**2**	**2**	**2**	**2**	**2**	**3**	**2**	**35–42**	**4000–4500**	**2**	**2**
RE51F	2	4	4	8	2	1	4	6	4	52	5800	2	2
RE51B	2	4	4	4	2	1	4	4	4	45	5200	2	2
RE52B	2	8	4	4	1	1	2	4	4	49	6150	4	2
RE53F	1	6	4	4	2	1	2	6	4	48	5000	2	2
RE53B	2	6	4	4	2	1	4	6	4	65	5300	2	2
RE54F	2	8	4	4	2	1	2	9	4	60	6500	2	2
RE54B	2	16	4	16	2	2	2	6	4	55	6550	2	2
RE55F	1	4	2	4	1	2	2	4	4	58	5500	2	2
RE55B	2	4	4	8	1	1	4	4	6	42	5200	2	2
RE56F	2	8	8	4	2	1	4	3	4	43	4800	2	4
RE57F	8	4	4	4	1	1	2	6	4	56	5200	2	2
RE57B	2	8	8	4	2	1	4	4	4	51	6000	2	2
RE58F	1	8	8	4	2	1	2	4	6	68	5500	2	2
RE58B	2	8	4	4	2	1	2	4	6	40	5950	2	2
RE59F	2	8	8	8	2	2	4	6	4	68	5100	2	4
RE60F	2	6	4	8	2	2	2	6	4	66	5800	2	2
RE60B	2	6	32	4	1	2	4	6	4	53	4550	8	2
RE61F	1	6	4	4	2	1	2	5	4	44	4900	2	2
RE61B	1	8	4	2	2	1	2	3	4	48	4750	2	2
RE62F	1	8	4	8	1	2	2	3	4	50	6700	2	2
RE63F	1	8	8	8	1	2	2	4	4	42	6150	4	2
RE63B	1	6	8	8	2	2	2	6	4	58	5000	2	2
RE64F	1	8	4	8	1	2	2	3	4	50	4100	4	2
RE64B	1	8	16	8	4	2	2	3	4	66	4300	2	2
RE65F	1	6	8	8	4	2	2	3	4	40	4500	4	4
RE65B	1	4	8	4	2	2	4	4	4	38	4000	2	2
RE66F	1	6	4	8	4	2	4	3	4	46	3000	2	4
RE66B	2	4	1	4	2	1	1	3	2	44	3800	2	2
RE67F	1	4	4	4	2	2	2	3	4	48	5700	2	2
RE67B	1	4	4	2	2	2	2	4	4	62	6000	2	2
RE68F	1	4	8	8	2	2	4	4	2	60	3500	2	4
RE69F	1	8	4	4	2	2	2	6	2	55	4000	2	2
RE70F	2	8	4	4	2	1	2	5	6	40	6500	2	2
RE70B	2	8	4	4	2	2	2	4	6	42	6800	2	2
RE71F	4	8	4	4	2	1	2	6	6	44	5700	2	2
RE71B	2	8	2	4	2	2	4	5	4	58	5600	2	2
RE72F	1	8	4	1	4	1	2	9	4	60	3500	2	2
RE73F	2	8	2	1	16	2	2	6	4	44	3900	2	2
RE73B	2	8	2	2	1	1	2	4	4	48	5000	2	2
RE74F	2	6	2	4	8	2	2	4	4	56	5100	2	2
RE74B	4	8	2	2	2	2	2	4	4	54	5200	2	2
RE75F	4	4	2	2	1	2	2	4	2	64	5500	2	2
RE75B	4	4	2	2	1	1	2	3	4	44	5000	2	2
RE76F	8	8	2	4	2	2	2	3	4	48	4500	2	2
RE76B	2	4	2	4	2	2	2	5	6	42	4200	2	2
RE77F	1	4	2	4	2	1	2	4	4	40	4800	2	2
RE77B	2	8	4	8	1	1	2	4	2	46	4500	2	2
RE78F	1	4	4	4	2	1	2	5	4	48	5800	2	2
RE78B	1	4	4	4	2	1	2	3	4	44	6000	2	2
RE82F	1	6	2	2	4	1	2	3	4	58	5300	2	2
RE82B	1	8	4	4	2	1	2	4	2	59	5400	2	4
RE83F	2	8	2	4	1	1	4	5	4	55	3700	2	2
RE83B	1	8	2	4	2	1	2	4	6	54	3500	8	2
RE84F	2	8	4	2	8	1	1	4	4	60	4800	2	4
RE84B	2	6	4	2	1	1	2	4	4	62	5000	2	4
RE85F	1	6	2	2	1	1	2	6	4	58	4200	2	2
RE85B	1	4	4	2	2	2	2	6	2	48	6550	2	2
RE87F	2	8	2	2	2	1	2	5	2	42	5300	2	2
RE87B	2	16	2	2	2	1	2	4	4	56	5600	2	2

# None of the NBR-exposed males contained normal copy number profiles for all the Y-linked genes studied.

### Multiple Polymorphic Copies of the *SRY* Gene and Exposure to NBR

Copy number of the *SRY* gene varied among the exposed males. Fathers and sons in several families showed 2 or more copies of this gene (Table 2). Of all the samples analyzed, ∼85% showed *SRY* copies ranging from 2–8. Irrespective of number of copies in blood DNA, germline DNA showed a normal single copy in 90% of exposed males.

Sequence analyses of PCR-amplified 20 *SRY* fragments (table 1) from each of the selected 100 exposed males showed insertions, deletions and silent point mutations scattered upstream, downstream and within the HMG box. Frequent transversions were noticed from pyrimidine (C/T) to purine (A/G) throughout the *SRY* exon. Approximately 90% of the insertions were of adenine or guanine only (table 3). Surprisingly, amongst multiple copies of *SRY* genes ranging from 4–8 detected in an individual, at least one copy remained normal with respect to its nucleotide sequence. This was in accordance with our previous study showing multiple copies of the *SRY* gene in one male 33F [19]. Multiple copies of the *SRY* gene have been reported in rodents [20], [21] but not in humans. Thus, in the present study, multiple polymorphic copies of the *SRY* gene correlated with the effects of NBR. The amino acid changes in some representative males are shown in Figure S6.

**Table 3:** Details of nucleotide and amino acid alterations in the *SRY* gene in some representative males exposed to NBR#.

S.N.	Seq ID	Point Nucleotide Changes	Amino Acid Changes
**1**	**RE53F**	Del A317, C410G	NO CHANGE
**2**	**RE54F**	Insertion T204, Insertion C363 and A381, T416C, C418T, A420C, G421A, Insertion G423, Insertion A800, T801, A219G, C231T,C468T	**T48M**
**3**	**RE55B**	Del A317, C410G, Insertion C332, Insertion C577	*DelL144–145*, *P146S*, *P149R*, *A150R*, *V152R*, *L153F*, *C154G*, *Del155–161*, *R162T*, *Y164Q*, *D166S*, *Del167–170*, *H173G*, *S174Q*, *R175Q*, *M176V*, *E177A*, *H178T*, *Q179G*, *L180M*, *G181T*, *H182V*, *L183R*, *Del184–204*
**4**	**RE55B**	C410G, T435G, A427T, C419T, Insertion C332, Insertion C577	*DelL144–145*, *P146S*, *P149R*, *A150R*, *V152R*, *L153F*, *C154G*, *Del155–161*, *R162T*, *Y164Q*, *D166S*, *Del167–170*, *H173G*, *S174Q*, *R175Q*, *M176V*, *E177A*, *H178Q*, *Q179G*, *Del180–204*
**5**	**RE57F**	Del A317, C410G	**H182T**, *P184T*, **P185A**, **I186H**, **N187Q**, **A188R**, **A189S**, **S190Q**, **S191L**, **P192T**, **Q193A**, **Q194A**, *R195T*, *D196G*, **R197T**, *Y198L*, **S199Q**, *H200P*, *W201L*, **T202D**, *K203R*, *L204A*
**6**	**RE57B**	Del A317, C410G	*K203R*
**7**	**R31F**	Del A317, C410G, Insertion C578 and 577, C192T, InsertionC541, A657C, A671C, C677A, G679C, A684C	**D166G**, **R165Q**, **Y164L**, **Del165–204**
**8**	**RE58F**	C410G, C419T, A427T, Del T435	NO CHANGE
**9**	**RE58B**	A427T	NO CHANGE
**10**	**RE60F**	C410G, T435G	**F12I**, **D17E**, **N24D**, **I25N**, **L28F**, **S32F**
**11**	**RE60B**	C410G, A427T, T435G	**Del187–204**
**12**	**RE65F**	InsertionC332	**DelL144–145**, *P146S*, *P149R*, *A150R*, *V152R*, *L153F*, *C154G*, **Del155–161**, *R162T*, *Y164Q*, *D166S*, **Del167–170**, *H173G*, *S174Q*, *R175Q*, *M176V*, *E177V*, *H178Q*, *Q179G*, **Del180–204**
**13**	**RE75F**	Del T82–83, Del336G, A596G, C733A, C808T	*Y44C*
**14**	**RE75B**	A140G, Del 336G,T458A,A670G	**S18G**, *Y44C*
**15**	**RE76F**	T226C, InsertionG235, T279C	*Y44C*, **D73K**, **Del49G-R72**, **Del75–204**
**16**	**RE80F**	InsertionC237, Insertion C365, Del T391, Insertion G397, Insertion A415, Insertion A448	*Y44C*
**17**	**RE80B**		*Y44C*, **Del84–85**, **R86Q**, **S88A**, **E89K**, **I90L**, **S91R**, **K92D**, **L94Q**, **Del95**, **Y96A**, **Q97S**, **K99D**, **M100T**, **L101S**, **T102G**, **E103K**, **A104C**, **E105Y**, **Del106–204**
**18**	**RE81F**	Insertion12C, 15G and 155T, A216G, InsertionT143, A228G, T467C, C535A, A495T, A573C, T636A, InsertionT638, InsertionC646, InsertionA/T670, C677A, C672G, A697T, G583T, C686A	**A20L**, **V21C**, **L35P E47G**, Y127H, K136M, **Y164H**, **R165M**, **L183I**, *P184T*, **InsertionA185**, **I187S**, **N188T**, **A190Q**, **A191P**, **K203N**, *L204A*, **F110L**, **N141D**
**19**	**RE81B**	Insertion12C, 15G and 155T, A216G, InsertionT143, A228G, T467C, C535A, A495T, A573C, T636A, InsertionT638, InsertionC646, InsertionA/T670, C677A, C672G, A697T, G583T, C686A	**A20L**, **V21C**, **L35P E47G**, Y127H, K136M, **Y164H**, **R165M**, **L183I**, *P184T*, **InsertionA185**, **I187S**, **N188T**, **A190Q**, **A191P**, **K203N**, *L204A*, **F110L**, **N141D**
**20**	**RE88F**	T188C, G292A, G313A	**no change**
**21**	**RE90B**	G371A	G95R, **Del152–160**, **N161G**, *R162T*, *Y164Q*, *D166S*, **Del167–170**, G95R
**22**	**RE94F**	T549C, T576C	**C154R**, *H173G*, **H174Q**, *R195T*, *D196G*, **R197P**, *Y198L*, **R199Q**, *H200P*, *W201L*, **T202V**, *L204A R175Q*, *M176V*, *E177V*, *H178Q*, *Q179G*, **Del180–204**
**23**	**RE94B**	G598T, C686A	**K203S**, **Del204**, **K43R**, **A171T**

# Amino acid changes in bold are random, underlined are reported in the literature, and italicized are consistently present in several exposed males.

### Expression Level of *SRY* Gene in the Exposed and Unexposed Males

**Figure 5:**
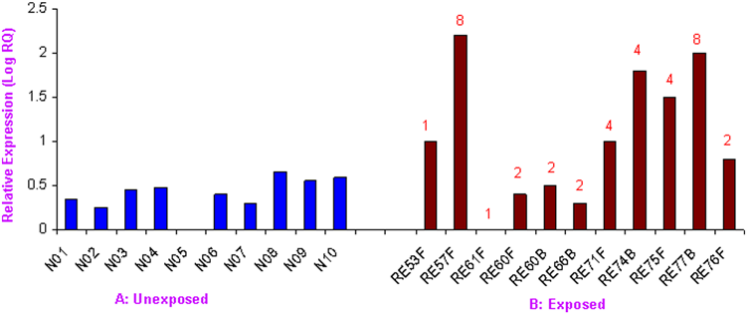
Expression of *SRY* gene in exposed and unexposed males based on real time PCR. (A) Unexposed males with single copy of *SRY* showing variable expression in lymphocytes. Transcript levels were normalized using β-actin and *RNase*P genes as internal controls, and a normal male (N05) as calibrator. (B) Exposed males showing fluctuating but conspicuously higher levels of *SRY* expression compared to that in normal ones, even with eight copies of the gene. The male RE61F was used as calibrator. Copy number of the *SRY* gene in corresponding males is given on top of each bar.

It is not possible to assess expression of Y-linked genes in human tissues or gonads owing to logistic constraints. Therefore, we studied expression of the *SRY* gene in the blood. Normal fertile males showed varying levels of *SRY* transcripts even among members of the same family within and across the populations. Similar variations were detected among exposed males, but in general, the expression level was several folds higher than in the unexposed ones (Figure 5). The difference in the expression level was conspicuous in males with the same number of copies. For instance, males 3B and 10B, with 2 copies of the *SRY* gene, showed 1.5–2 times the level of expression, whereas 9F, 10F, 31F, 32F, 33F and 33B, also with two copies, showed higher levels (around 4–9 times) of expression. Males 29F and 29B, with four copies of the *SRY* gene, showed similar levels of expression as those having 2 copies. For calculations, a male (8F) with the lowest level of *SRY* transcripts was used as a calibrator in real time PCR. Startlingly, males with 8 copies of the *SRY* gene (e.g., 7F) showed 25 times higher expression as compared to that of the calibrator.

### Multiple Polymorphic Copies of the *CDY1* Gene and Background Radiation Exposure

**Figure 6:**
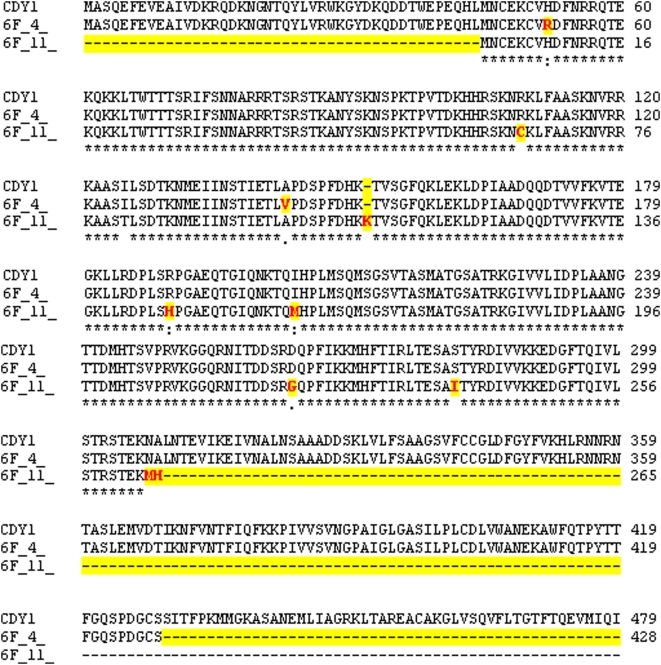
Two different copies (6F_4, 6F_11) of *CDY1* gene in NBR-exposed male (6F) are shown along with its normal sequence (top). Note the conspicuous difference between the two copies. Amino acid changes corresponding to nucleotides are highlighted in red with yellow background.

Sequencing of full length *CDY1* genes (primers in Table 1) showed polymorphism similar to that detected in *SRY* genes. The *CDY1* gene from a single male (6F) was amplified, cloned into *pGEMT* easy vector, and sequenced (Figure S7). Nucleotide analysis of 5–10 full-length *CDY1* fragments, each from 50 exposed males, uncovered random point mutations throughout the single exon. All the exposed males showed two types of *CDY1* sequences, one full length with inserted intron, and the other one with point nucleotide changes leading to correspondingly truncated *CDY1* protein as uncovered *in-silico* (Figure 6). Once again, this highlighted polymorphism of the multiple copies of *CDY1* gene, similar to that of *SRY*. Sequence comparison of the *CDY1* gene from representative exposed males is given in Figure S8. Based on our data, we inferred that NBR exposure leads to the formation of multiple polymorphic copies of the Y-linked genes. This was substantiated by full length sequencing of the *XKRY* gene showing nucleotide changes spread all across its length, though frequently in the middle portions (not shown). Although there is no report on the implications of this gene for male fertility, its small size makes it an attractive candidate for the assessment of point nucleotide changes.

### *DAZ* Genes (*DAZ*1–*DAZ*4) in Males Exposed to NBR

Analysis of SFV/SNV/STS in 390 exposed males showed all the six intact SNVs, confirming the presence of four *DAZ* genes in >85% of males. Similar SNV/SFV analysis of the remaining 15% showed either the absence of an allele, or absence of a single band corresponding to a specific SNV (Table 4). Another intriguing observation was the loss of the restriction enzyme site for SNVII since the PCR product remained undigested in >55% of exposed males. However, the majority of the NBR-exposed males showed intact *DAZ* genes.

**Table 4:** AZFc region SNV/SFV typing in males exposed to NBR#.

SNV	Haplotypes	Samples	Number
***DAZ*** ** SNVs**			
I	*A(709)*+*B(398)*	RE3, RE7, RE8, RE9, RE40, RE41, RE52, RE53, RE56, RE61, RE109, RE110	12
II	A+B	+ve in all	
		PCR amplicon not digested in more than 55% of the males	
III	A+B	+ve in all	
IV	A+*B(122)*	RE5, RE6, RE10, RE11, RE18, RE23, RE25, RE27, RE40, RE47, RE55, RE57, RE59, RE81, RE89, RE105–RE110	20
V	*A(195)*+B	RE111, RE122, RE123, RE124, RE127, RE143, RE144, RE146, RE147, RE157, RE158, RE159, RE167, RE168, RE170, RE172, RE173, RE189, RE190, RE191, RE197, RE200, RE201, RE202, RE203, RE205, RE206	27
**AZFc SNVs**			
AZFc-P1/1	A+B	+ve in all	
GOLY/1	**A**+*B (298)*	+ve in more than 90% of males	
BPY2/1	A+**B**	Allele B absent in ∼15% of males	
TTY4/1	**A**+B	167 out of 390 males	167
RRM3	+ve in all		
Y-DAZ3	−ve in 60% of males		180

# The *italicized* haplotypes showed absence of a single band (size given), and the one shown in **bold** was absent.

### Status of the Other *AZFc* Candidates

**Figure 7:**
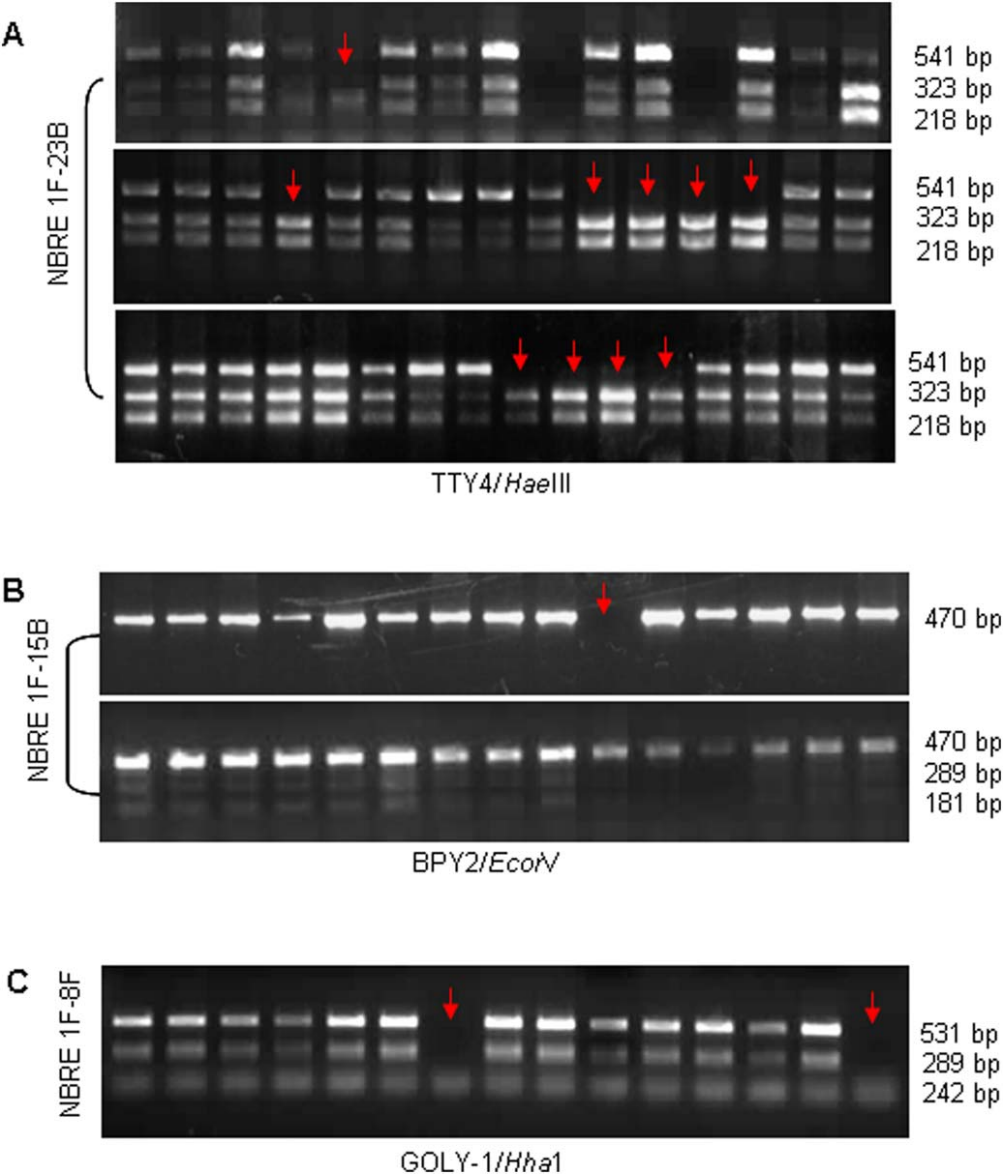
AZFc SNV/SFV typing in the exposed males. “NBRE” refers to natural background radiation–exposed males, and numbers represent the family IDs. (A) Loss of SNV *TTY4/1* allele “A” (541 bp) in exposed males (arrows in panel “A”). Sizes of digested DNA with *Hae*III enzyme are given on the right. Note the absence of 541 bp band in several males. Some showed complete absence of the SNV *TTY4/1*. (B) SNV for *BPY2* gene showing its absence in some males (arrow) and loss of allele B (289+181 bp) in others (upper panel). (C) SNV typing of *GOLGA2LY* gene. Note the absence of allele “A” and 289 bp of allele “B” in some males (arrows in “C”).

SNV/SFV analysis of the other *AZF*c candidates revealed complete absence of STS Y-*DAZ*3 in blood DNA in approximately 60% of the exposed males (Table 4). The TTY4/1 allele A was absent in 167 of the 390 males. Similarly, allele B of the SNV BPY2/1 was absent in 15% of males, Likewise, allele A and a 398 bp fragment of allele B of the SNV GOLY/1 were absent in >30% of exposed males (Figure 7). The other *AZFc* SNVs *AZFc*-P1/1 and RRM3 were normal in all the cases.

### Tandem Duplication of the Y-Linked Loci in Males Exposed to NBR

**Figure 8:**
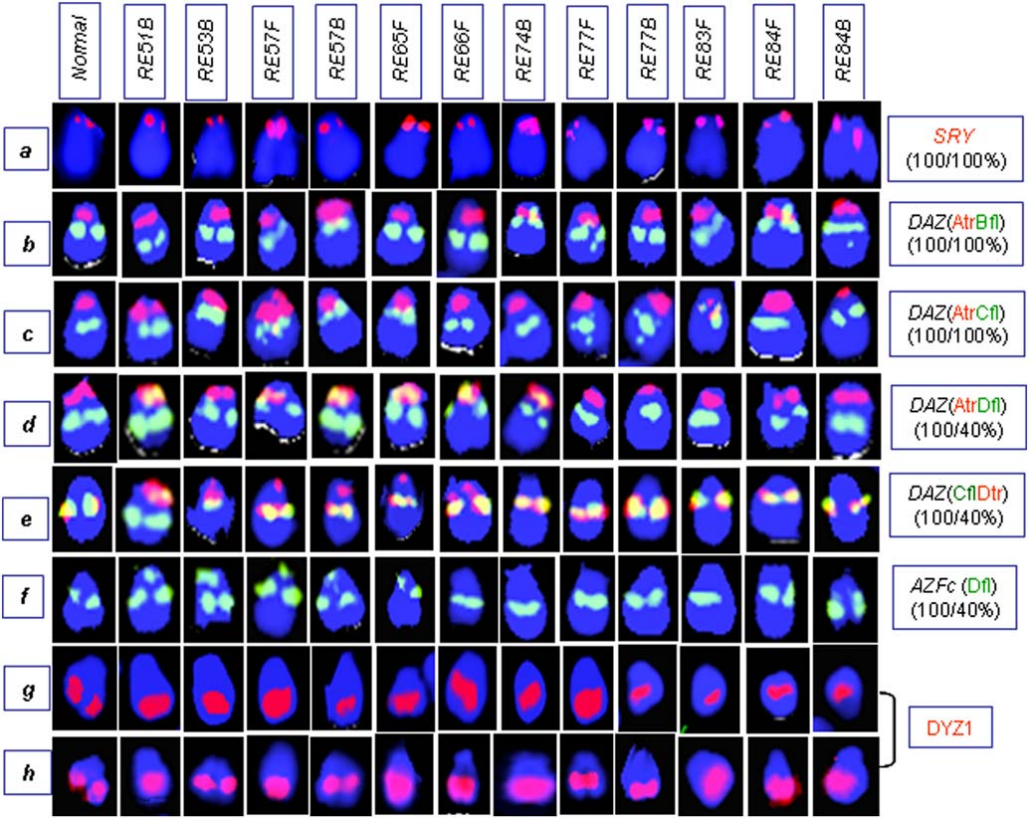
Structural organization of the human Y chromosome in exposed males. Probe combinations are described in table S4. The abbreviations “*fl*” and “*tr*” refer to fluorescein and Texas red labels, respectively. Probe combination A*tr*B*fl* corresponds to dual color FISH with 18E8 in red and 63C9 in green. (a) *SRY*-FISH showing a single signal with varying intensities, with its multiple copies representing tandem duplication. (b) FISH with A*tr*B*fl* showing localization of probe “A” onto the proximal Yp region in all the NBR-exposed and normal males. This was substantiated using A*tr*C*fl* and A*tr*D*fl* probe combinations shown in (c) and (d), respectively. FISH with probe “D” showed exclusive localization of one of the three green amplicons onto the short arm, overlapping with the signal for probe “A”, which was substantiated using probe combinations A*tr*D*fl*, D*tr*C*fl*, and D*fl* shown in (d), (e), and (f), respectively. Such localization was detected in 85–90% of exposed males. Percentage of cells showing probe D signal on the short arm was higher in the exposed males compared to unexposed ones. The ratios show percent of males to percent of cells (e.g., in probe D, panel f, 100/40% means only 40% cells of all the males showed alteration with respect to probe “D”). (g–h) Variation in length and position of Yq heterochromatin detected using 3.4 kb repeat unit of DYZ1 as FISH probe. This fluctuation was more prominent in the exposed males compared with an almost uniform signal in normal males (NM).

Fluorescence *in situ* hybridization (FISH) conducted on metaphase chromosomes and interphase nuclei of the normal males showed a single signal in each of the *SRY* and *DXZ1* probes [19] (Figure 8a). As expected, a single signal was detected in the centromeric region of each X chromosome in the normal female used as negative control (data not shown). Exposed males also showed a single signal with multiple copies of the *SRY* gene. A male (7F) carrying eight copies of *SRY* showed a relatively stronger signal on the Y chromosome compared to that of a normal male. However, in several cases with 2–4 copies of *SRY*, differences in the signal intensity were not discernible. Depending upon the condensation status of the sister chromatids in some cases, two signals of the *SRY* were seen. Interestingly, ∼5–12% of males lacked signals for the *SRY* gene, whereas a signal for *DXZ1* was consistently detected. The absence of signal was attributed to the loss of Y chromosome shown in the later section.

**Figure 9:**
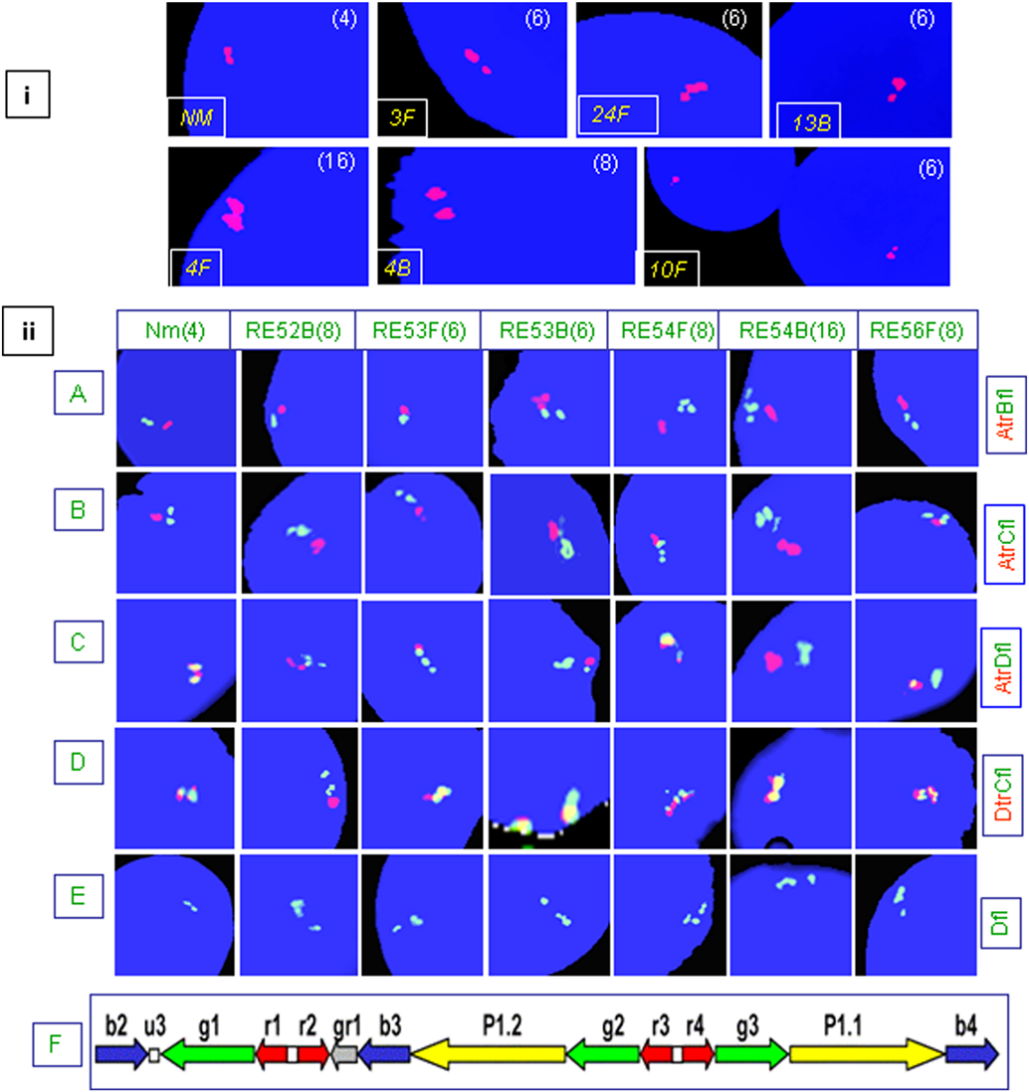
(i), Tandem duplication of *DAZ* genes in NBR-exposed males uncovered by FISH. Numbers in parentheses represent copies of *DAZ* genes ranging from 4–16 in the exposed males with corresponding variation in signal intensity. Sample IDs are given in square brackets. Note three signals in samples 3F, 24F and 13F, with two on one side and a single one on the other, highlighting the unilocus duplication of *DAZ* genes (see text for details). (ii), Organization of *DAZ* genes in exposed males. Only six representative exposed males and a normal one (Nm) are shown, with their sample IDs on top, copies of *DAZ* in parentheses, and probes on the right of the panels. The normal male (Nm) showed the expected number of discernible signals for each probe (A–E) with combinations of A*tr*B*fl* and A*tr*C*fl* separating two signals. Exposed males showed higher *DAZ* copies corresponding to unilocus (RE53F & RE53B) or bilocus duplications (RE52F, RE54F, RE54B, & RE56F) evident from the number and intensity of the corresponding signals shown in (A), (B) and (D), respectively. Similarly, probe “D” specific for green amplicons showed three expected signals overlapping “B” with “C” in normal males. In exposed males, 80–90% of cells showed unexpected overlap of probe “D” signal with that of “A” (RE53F, 54F & 56F), as shown in (C). Presence of three signals with probe D was confirmed by different probe combinations as shown in (D) and (E). (F) Schematic map of the *AZFc* amplicons given as a reference.

FISH probes (Cosmids 18E8, 46A6 and 63C9) used for the *DAZ* genes have been explained in the literature [22]. Cosmid probe 18E8 encompassing the 5′ end of two neighboring *DAZ* genes showed two signals in the normal males, or one, if the same were merged. In 20% of exposed males with 6 copies of *DAZ* genes, three signals were discernible (Figure 9i). In these exposed males, only one locus of *DAZ* underwent duplication (unilocus duplication), resulting in six copies. Males with 8 or 16 copies showing correspondingly stronger signals compared to that of normal males seem to have undergone single and double rounds of tandem duplications, respectively (Figure 9). Except for males with unilocus duplication, approximately 30% showed three signals in about 9–12% of cells (not shown) and complete absence of signals in 10–15% of interphase nuclei (explained later). The absence of *DAZ* signals was construed to be due to the loss of Y chromosome. This inference was based on a number of Y-specific probe combinations and painting described in later sections. As expected, the mosaicisms affected Ct values of *DAZ* and *SRY* genes during real time PCR assay.

### Structural (Re)organization of the *DAZ* Genes

**Figure 10:**
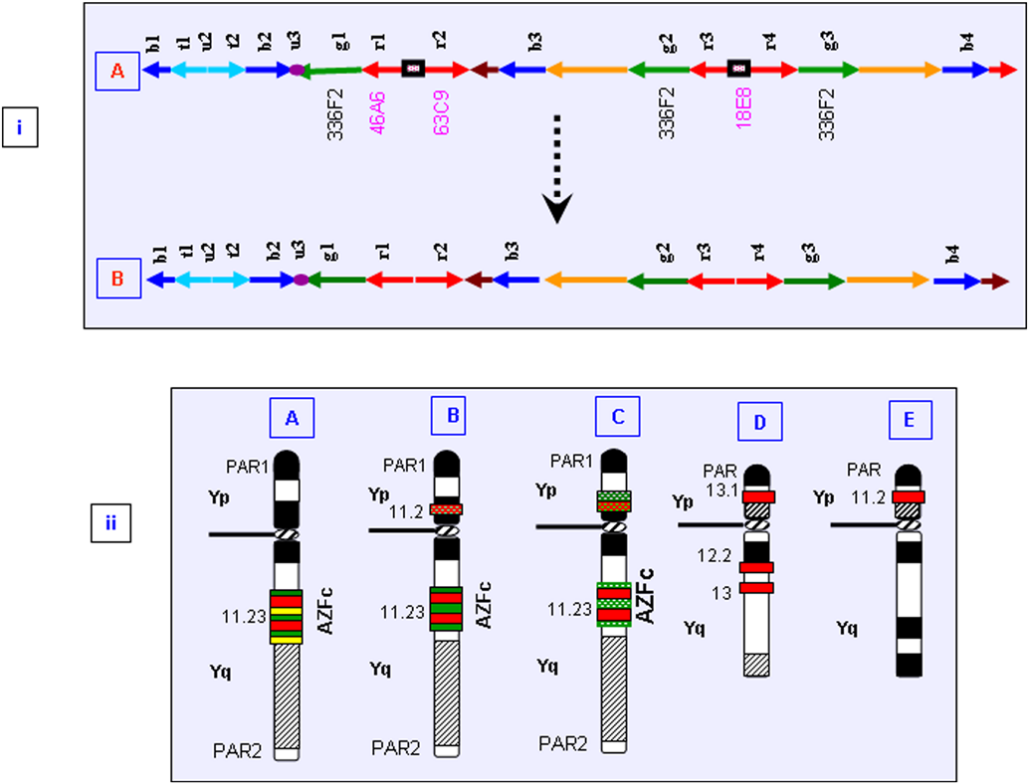
(i), Schematic representation of the *AZF*c region. (A) Arrangement of the *AZFc* amplicons as reported in the literature. Probes used in this study are given below the corresponding amplicons. Cosmids common to all the *DAZ* genes are highlighted in pink. (B) Arrangement of the *AZF*c amplicons in the NBR-exposed and control males showing decreased intervening length between the two *DAZ* genes at each locus. This decrease is hypothesized to be due to translocation of 18E8 sequences onto the Yp. (ii), (A) Diagrammatic illustration showing positions of *DAZ* genes and neighboring *AZF*c amplicons reported thus far. Red horizontal bars represent two *DAZ* loci. The amplicons *g1*, *g2*, and *g3*, detected with BAC 336F2 (probe D) are represented by green horizontal bars. (B) Translocation of probe “A” signal to the Yp shown as red dotted horizontal bar. (C) Duplication of either one or all the *g1*, *g2*, and *g3* amplicons followed by translocation to the short arm with higher frequency in NBR-exposed males compared to that in normal ones. (D) and (E) represent Y chromosomes of Sumatran orangutan and pygmy chimpanzee, respectively, where *DAZ* genes are present on the short arm of the Y chromosome. The Yp localization of *DAZ* genes was not linked with the exposure to NBR, since a similar arrangement was detected in the normal males.

Previously, two *DAZ* loci in the *AZFc* region, each with an inverted pair of *DAZ* genes (amplicons *r1*, *r2*, *r3*, *r4*) and an inter-*DAZ* sequence were detected. We observed a similar arrangement in the interphase nuclei of the exposed males. But subsequent hybridization of *DAZ* probes with metaphase Y chromosomes revealed a different organization. The cosmid 18E8 (probe A) representing 5′ *DAZ* exons 1 through 7 on one side and the inter-*DAZ* region on the other [22] was localized onto the proximal Yp instead of on the anticipated Yq region in all 390 exposed males (Figure 8). The other two *DAZ* cosmids 46A6 and 63C9 (probes “B” and “C”) representing *DAZ* exons 2 through 11 and 3′ *DAZ*, respectively, were localized on their anticipated positions in the Yq region, overlapping with each other on the metaphase Y chromosome (Figure 8). Similar results were obtained with FISH conducted on interphase nuclei (Figure 9ii). Signals for probes “A” and “C” or “A” and “B” never overlapped. Another probe (D) corresponding to neighboring *g1/g2/g3* amplicons of the *DAZ* genes detected the expected 3 signals on the interphase nuclei. On the metaphase Y chromosome, probe “D” detected the expected overlap with probes “B” and “C”. In addition, in each of 80–90% of exposed males, 70–80% of cells showed an unexpected overlap of probe D signals with that of probe A on the proximal short arm of Y chromosome (Yp). Overall structural reorganization of the *AZFc* region and *DAZ* genes in the exposed males is summarized in Figure 10. Similar (re)organization was detected in normal males and thus this was not attributed to the effect of NBR.

### Long Arm Heterochromatin of the Y Chromosome (Yqh)

Prominent length variation of the Yq heterochromatin was detected in the exposed males using a 3.4 kb FISH probe (Figure 8g–h) compared with an almost uniform signal in the normal males. This variation was construed to be due to copy number variation (CNV) of the 3.4 kb repeat units. In normal males, the average number of copies of the DYZ1 repeat varies from 4000–4500. In the exposed males, this number was detected in the range of 4500–6500 (table 2). The increase in DYZ1 copy number among 390 males correlated with varying FISH signals. Interestingly, ∼5–12% of cells per exposed male were devoid of signal, suggesting loss of Y chromosome.

### Loss of the Y Chromosome and NBR Exposure

**Figure 11:**
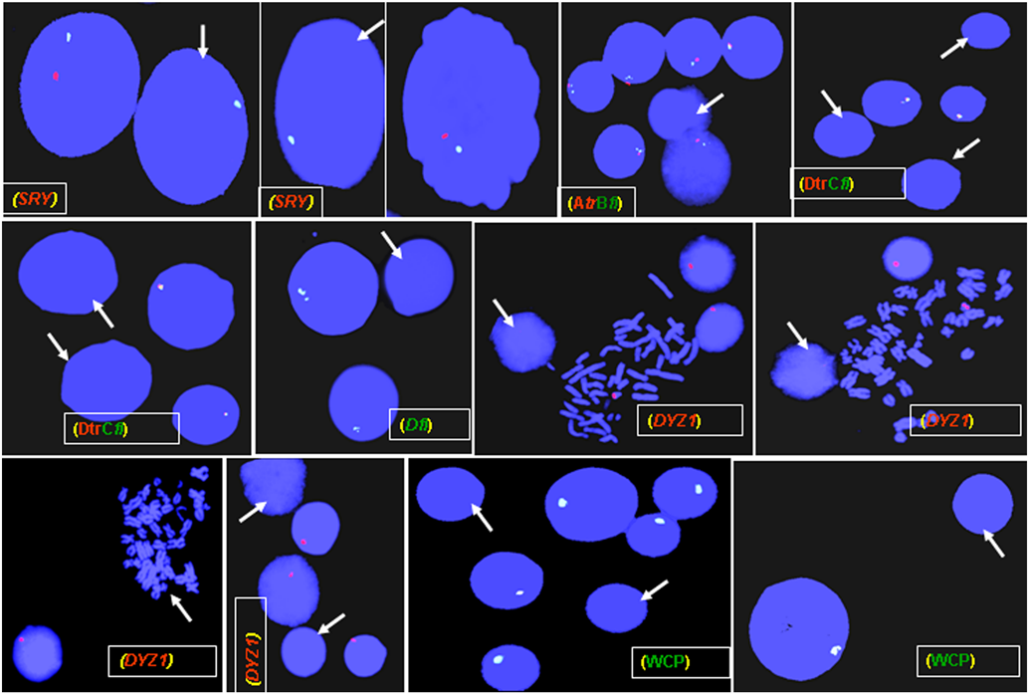
Loss of Y chromosome in 10–15% of cells in NBR-exposed males. Details of probes and probe combinations labeled with red (Texas red, *tr*) or green (fluorescein, *fl*) are given in parentheses. Probe symbols (A–D) are the same as given in Figures 1 and 2. *SRY* probe combination hybridizes simultaneously to *SRY* gene on the Y (Yp11.3) and centromeric region of the X chromosome (DXZ1). WCP refers to whole chromosome painting showing loss of the Y chromosome (arrows).

Conclusions on FISH were drawn based upon the analysis of 400 interphase/metaphases each from 390 exposed males. As mentioned earlier, FISH conducted with *SRY/DXZ1* showed DXZ1 signals on the X-centromere in all the cells, but *SRY* signal was missing in 5–12% of cells (Figure 11). Similar observations were made using different *AZFc* and *DAZ* probes individually and in combination with one another. Whole chromosome painting confirmed the loss of Y chromosome as mentioned earlier (Figure 11). In normal males, ∼96–98% of cells were positive for all the probes whereas in the remaining ones, signals were not detected owing to technical constraints.

## Discussion

Owing to its haploid status, repetitive nature and intrachromosomal homologous recombination, the human Y chromosome is highly prone to genetic variations [23], [24], which are passed on to the next generation un-repaired. However, this chromosome has been implicated with male sex determination and spermatogenesis. Thus, lethal mutations are usually not transmitted to the next generation. The present study is an attempt to explore genetic variations of the human Y chromosome in males exposed to NBR.

### The *AZF* Microdeletions and Radiation Exposure

Ionizing radiation induces several types of DNA lesions through single-strand (SSB) and double-strand (DSB) breaks, AP sites (either apyrimidinic or apurinic), and DNA–DNA and DNA–protein cross-linking, in addition to base modifications [25], [26]. Several alterations in the DNA induced by ionizing radiation are chemically identical to those caused by reactive oxygen species [27]. Ionizing radiation induces isolated as well as clustered damages involving two or more lesions within one or two helical turns of DNA. Base lesions within the clustered DNA enhance biological severity of the damage. Highly localized DNA damage caused by ionizing radiation leads to intrachromosomal breaks [28], [29]. Several attempts have been made to establish a correlation between background radiation, chromosomal aberrations and phenotypic changes [5]–[8]. However, due to insufficient data, experimental evidence of radiation induced mutations in humans still remains highly controversial.

Our study demonstrated a correlation between radiation exposure and microdeletions in the *AZF* regions. These microdeletions did not follow a normal pattern of inheritance since deletions detected in fathers were absent in their sons and vice versa. This discrepancy, observed in most of the exposed males, was true for gene copy number variations as well. This can be explained on the basis of unaffected germline DNA in the exposed males. We postulate that radiation exposure caused localized DNA damages but by some unknown protection mechanism, the germline DNA remained intact. Scrutiny of microdeletions showed higher frequency in proximal and distal *AZFc*, and to some extent in the *AZFa* region, but absence of any major deletion phenotypes such as *gr/gr*, *b1/b3*, *b2/b4* (*AZFc*) and HERV mediated recombination (*AZFa*). Absence of microdeletions in the germline explains the normal fertility status of the exposed males.

Somatic (blood DNA) deletion of the *DBY* gene observed in 25% of exposed males was intriguing since such deletion has been correlated with male infertility [30]. However, normal fertility in the exposed males is maintained because of the intact *DBY* gene in the germline. The negligible frequency of microdeletions detected in normal males may be taken as internal polymorphisms of the Y chromosome. In view of the microdeletions absence of major deletion phenotypes and normal fertility of the exposed males, diagnosis of the infertile males on the basis of microdeletions may prove to be inadequate. Instead, the patients may be subjected to selection criteria, ethnic variations and detailed experimental design to pin-point involvement of Y-microdeletions during spermatogenesis. In addition, levels of the hormones, quality of the semen samples and sperm counts may also be taken into consideration. This is important because in certain ethnic groups, absence of a pair *of DAZ* gene does not seem to affect fertility status of the males [13].

### Gene Duplications and the Human Y Chromosomes

CNPs in humans have been reported using genome wide array-CGH techniques [31]–[34]. But these approaches have limitations due to the limited number of samples taken up for analysis and low resolution. This problem can be circumvented by using TaqMan probes, which differentiate between a single copy *SRY* and *RNase*P genes in blood and semen DNA samples. The human genome contains blocks of duplicated sequences which are prone to rearrangements resulting in genomic variation [32], [35]. CNPs of gene(s) related to disease resistance or susceptibility, responsiveness to a particular drug, and aging ailments have been reported [34]. Despite its wide-spread occurrence in the human genome, no report is available on the Y-linked genes. Thus, the present report on CNP of the Y-linked genes seems to be a singular observation which is well within the realm of genetic variation and genome evolution. Gene duplication increases the complexity of a genome, particularly when it is not clear if the resultant copies follow normal levels of expression [36]. Incidentally, this question cannot be resolved by Southern blot hybridization [37]. The presence of 4–16 copies of the *SRY* gene detected in the NBR-exposed males demonstrated that non-disjunction of the Y chromosome and duplication of Y-linked genes are independent events.

### Tandem Duplication of the Y-Linked Genes

The localized FISH signals of the candidate *SRY* and *DAZ* genes on the interphase nuclei and metaphase Y chromosomes in all the males exposed to NBR confirmed their tandem duplication. Earlier studies demonstrated two *DAZ* clusters, each harboring an inverted pair of *DAZ* genes separated by a 40–43 kb intervening sequence [22]. We detected translocation of this intervening region from Yq onto the proximal Yp in more than 95% of cells from all the exposed and unexposed males using cosmid 18E8 [22]. This suggested that the structure of the Indian Y chromosome is much different compared to the one reported in the literature. This issue has been discussed in detail in a separate manuscript.

### Copy Number Variation (CNV) and Sequence Polymorphisms

Identification of several new players in the sex determination pathway [38] indicates that there is lot more to explore in terms of the actual role of the *SRY* gene. This view was substantiated by the analyses of this gene in exposed males (present study) and SCRA patients [19]. Most interesting was the duplication of this gene not reported earlier. A classical model suggests that after gene duplication, one copy preserves the ancestral function while the other one may conform to a new function [39]. Alternatively, another model, duplication, divergence and complementation (DDC), suggests that duplicated genes are preserved because each copy loses some, but not all of its functions through degenerating mutations [39]. Gene duplication and alternate splicing are inversely correlated evolutionary mechanisms fuelling the process of new biological functions [40]. NBR might have initiated the process of gene duplication leading to CNV. Sequence polymorphism, CNV, and the presence of only one normal copy of the *SRY* gene suggest that the hidden functionality of such a phenomenon only becomes apparent when the genome is challenged by environmental factors/stresses. Thus, tandem duplication of genes detected in the present study possibly occurred in order to absorb the mutation load caused due to NBR. We observed males with higher copies of *SRY* genes showing correspondingly enhanced levels of *SRY* transcripts. However, it was not clear if all the transcripts were translated. In the presence of abundant mRNA transcripts, it is logical to construe that transcriptional machinery failed to discriminate between normal and altered versions of a duplicated gene. Tandem duplication of *SRY* followed by its augmented transcription may well be a possible mechanism of up-regulation of a gene.

Polymorphism of multiplicated Y-linked genes became more evident by sequence analysis of the *SRY* in other exposed males showing several known and novel mutations. Besides *SRY*, sequence polymorphism was observed in *CDY1* and *XKRY* genes, supporting the hypothesis that in the exposed males, other Y-linked genes are also affected. This was corroborated by SNV/SFV typing of *DAZ* genes in males exposed to NBR. These males showed either complete absence of a particular SNV allele or presence of additional amplicons. Similar to *DAZ* SNVs/SFVs, the *AZFc* region also showed polymorphisms. All these somatic sequence variations in the males correlated with the NBR exposure.

### Possible Mechanisms of Gene Duplication

A single copy gene gives rise to a number of mRNA transcripts, some of which may be reverse transcribed and transposed back into the DNA, resulting in multiple copies [41]. Thus, besides putative tandem duplication, reverse transcriptase activity on the mRNA may also contribute to CNV. However, this seemed to be a less likely event because *SRY* copies detected were in multiples of two to a maximum of 16. Owing to the availability of a large number of mRNA transcripts, reverse transcriptase–like activity would have generated far greater numbers of *SRY* copies.

### Somatic Nature of Alterations in NBR-Exposed Males

Natural background radiation may be responsible for varying alterations. However, intact germline DNA of the exposed males suggested a strong protective mechanism to counter the effects of NBR. Alternatively, it is likely that owing to high turnover of meiotic activities, defective cells undergo rapid apoptosis leaving behind no trace of an alteration in the germline. We are aware of the fact that, based on the analysis of some Y-linked genes, no major conclusion on the effect of NBR can be drawn. Thus, more genes involved in control and regulation of signal transduction, apoptosis, genome imprinting, tumor suppression and progression may be included to monitor the overall impact of NBR on the genome. This would go a long way towards undertaking corrective measures to save the population at high risk, if any.

### Conclusions

The present study demonstrates the effects of NBR on gene duplications and sequence polymorphisms, a possible genome strategy to absorb the mutational loads. This is evident from the multiple polymorphic copies of the *SRY* and *CDY1* genes. Moreover, from the enhanced *SRY* expression in blood, it is logical to construe that transcriptional machinery failed to discriminate between normal and altered versions of a duplicated gene. Absence of microdeletions and CNV of genes in germline DNA of exposed males suggested that the germline DNA is protected by some innate, still unexplored repair mechanism(s). Analysis of the additional Y-linked loci, and even the autosomal genes, may uncover the overall impact of NBR on the structural and functional attributes of the human genome.

## Materials and Methods

### Sample Collection

Peripheral blood and semen samples were collected with informed consent from 390 males of two generations at Chavara (lat. 8°57.8′N, long. 76°31.8′E) and Thevara (lat. 9°58′N, long. 76°16′E) in Kerala, India [17]. This study was reviewed and approved by the Institute's Ethical and Biosafety Committee. Since the population at the site of the NBR sample collection is illiterate, oral consent was obtained from the participants, facilitated by a recognized local clinician. In the case of control samples from other parts of India, written consent was obtained wherever possible. After due scrutiny, the clearance by the Institute's Ethical Committee was accorded. Following this, no additional approval regarding a particular gene sequence/gene variant was required for publication of the data.

The semen analysis of the exposed males was conducted by a commercial technician and the results are given in Tables S1 and S2. For control, blood and semen samples from 390 healthy unexposed males of matched age groups from Kochi city (South India) and 400 normal males from different parts of India were included. DNA from blood and semen was isolated following standard protocols. RNA was isolated from lymphocytes separated from blood with Accuspin™ System-Histopaque®-1077 (SIGMA, cat no: A7054) using TRI-X reagent (MRC, cat no: TB-126-200) and cDNA synthesized using high cDNA Archive Kit (cat no: 4322171, ABI, USA).

### STS Mapping of the *AZF* Regions

The *AZF* regions were screened in blood and semen DNA from normal and exposed males for the presence or absence of STS markers, and details of the primers used are given in Figures 2 and 3. The *AZF*c region was assessed for the intactness of the sites *P1.1/P1.2*, *GOLGA2LY*, *BPY2* and *TTTY4* genes by the STSs and SNVs [12], [13] (Table S3). PCR was carried out in 100 µl, precipitated with Na^+^-acetate/ethanol and used for restriction digestion with the recommended enzymes. The digested DNA was resolved on 2–3% agarose gel. For human endogenous retrovirus (HERV) repeats, sY82, sY746, sY1064, sY86 sY85, sY84, sY1065, sY1066, and sY88 STSs were used [23].

### PCR Amplification and Sequencing of the *SRY* and *CDY1* Fragments

The *SRY* and *CDY1* genes were amplified from semen and blood DNA with primers given in Table 1. Approximately 20 fragments amplified with each primer set from each male were sequenced. Sequences were analyzed by NCBI Blast and ClustalW alignments. Some PCR amplicons were cloned into *p-GEMT* easy vector (Promega, USA) for subsequent use.

### Silver Staining of the DNA in Polyacrylamide Gel

For small sizes of *AZFc* haplotype specific bands in the SNV/SFV typing, a 12% polyacrylamide gel was run in 1XTBE and silver stained for band visualization. Gel was treated with fixative-1 (40% methanol, 10% acetic acid) for 30 min followed by 2 washes with fixative-2 (10% ethanol, 5% acetic acid) for 15 min each. Gel was then rinsed in the oxidizer (1% potassium dichromate, 0.2% HNO_3_) for 5 min followed by 3 washes of de-ionized water until all the yellow color disappeared from the gel. Following this, gel was treated with 2% AgNO_3_ for 20 min and then washed with de-ionized water again until the brown precipitate was removed. Finally, gel was rinsed in developer (3% Na_2_CO_3_, 0.05% formaldehyde) for about 5 min or until the bands appeared. The action of developer was stopped by treating the gel in a stop solution (5% acetic acid) for 5 min.

### Primers, TaqMan Probes, and Conditions for Real Time PCR

For real time PCR, “Assay on Demand” specific for *SRY* gene (ID: Hs00243216_s1, nucleotide location 453) and an endogenous control, *RNase*P gene (single copy per haploid genome; Catalog number: 4316831), having FAM/TAMRA and FAM/MGB probes, respectively (Applied Biosystems, USA), were used. The TaqMan assay for *DAZ* genes was based on a 100 bp fragment of the sY587 sequence. Similarly, TaqMan assays were designed for other candidate genes by selecting a unique fragment for each one of them. Primers and FAM/NFQ probes were designed by “Primer Express Software (v 2.0)” and procured commercially from the “Assay on Demand” service of Applied Biosystems (ABI, USA). TaqMan universal PCR Master Mix (P/N: 4304437), standard male genomic DNA (P/N: 4312660), and a kit (P/N: 4316831) having FAM/MGB probe for *RNase*P gene and CEPH family female genomic DNA were purchased from ABI, USA. Using male genomic DNA and primer for *RNase*P gene, TaqMan assays were conducted. All the reactions were run in triplicate on the real time PCR (Sequence Detection System, 7000, ABI, USA). The efficiency and specificity of each TaqMan Assay were established using 10-fold dilution series of the standard male genomic DNA. Female genomic DNA was used as a negative control. Temperature profile for PCR reaction included 50°C for 2 min, 95°C for 10 min, and 40 cycles of 95°C for 0:15 min and 60°C for 1 min (referred to as universal PCR conditions by ABI, USA). Approximately 10–15 ng of genomic DNA per reaction, per individual from blood and semen samples were used. Copies of the genes were calculated by comparative Ct method in an absolute quantitation assay following standard protocol [19]. All the samples were used in triplicate and reactions were repeated at least three times to ensure error free consistent ΔCt values. The haploid status of Y-linked genes in comparison to autosomal *RNase*P in case of DNA from blood was taken into consideration for copy number calculation. The details of the genes, respective TaqMan assays, and target sites of the TaqMan probes are given in Table 1.

### Copy Number Estimation of the Y-Linked Genes and Expression of the *SRY* gene

Copies of the genes were calculated using the formula: Copy Number = (1+E)^−ΔCt^, where E is the efficiency of the PCR and ΔCt = difference in threshold cycle value between the test sample and endogenous control. If the efficiency is maximum (one), the copy number of test gene is  = 2^−ΔCt^. The relative level of *SRY* transcripts in blood was calculated by the ΔΔCt method following standard protocol (ABI). In this case, Ct values were normalized twice, first with the *RNase*P gene as an endogenous control and second with the sample showing highest Ct value (lowest expression) using SRY gene as calibrator sample. All the reactions were conducted on Sequence Detection System-7000 as mentioned above (ABI, USA).

### Copy Number Calculation of *DYZ*1 Repeat

Copy number of DYZ1 was calculated based on absolute quantitation assay using SYBR green dye and Sequence Detection System-7000 (ABI, USA). A set of primers specific to DYZ1 (DYZ1F- 5′ TGGAATGGAATCGAATGGAATGGAA 3′ and DYZ1R- 5′ TGCCAAATCATTGCATTCCTTTCC 3′) was designed using Primer Express Software V2.0 (ABI). The efficiency of the primers was assessed using a 10-fold dilution series of the recombinant plasmid and standard male genomic DNA. All the PCRs were repeated thrice in triplicate. Copies of the DYZ1 arrays were calculated by extrapolation of the standard curve obtained with known copies of the recombinant plasmid.

### Southern Hybridization

Microdeletions present in *AZF* regions were confirmed by Southern hybridization using 200 ng of genomic DNA and [^32^Pα-dCTP] labeled PCR product as probe. Deletion of *DBY* gene was confirmed following similar protocol of Southern hybridization using 50 µg genomic DNA.

### Fluorescence *in situ* Hybridization (FISH)

Approximately 400 µl of whole blood was cultured for chromosome preparation following standard protocols [19]. LSI *SRY* (Cat 32-191007) and WCP Y Spectrum green (Cat 32-122024) DNA FISH probes for *SRY*/CEP X and Y chromosome, respectively, were purchased from VYSIS (Illinois, USA). Cosmid probes 18E8, 63C9 and 46A6 were purchased from the Gene service, UK (www.geneservice.co.uk/home). BAC clones were purchased from the Children's Hospital Oakland Research Institute (CHORI), and FISH was conducted following standard protocol [19], [22]. Biotynilated anti-fluorescein and anti-Texas red antibodies coupled with fluorescein and Texas red avidin DCS (Vector Labs) were used in dual probe FISH experiments. Details of all the FISH probes are given in Table S4.

## Supporting Information

Figure S1 STSs amplifying additional bands. The ‘M’ is the molecular marker, ‘RE’ is radiation exposed, ‘N’ is normal unexposed males and, ‘F’ and ‘B’ denote father and son, respectively whereas the numbers state the respective families. Note the additional bands in case of sY1201, and sY1206 where the normal unexposed males showed only a single expected band. Similarly, in case of sY84 and sY86, multiple bands were observed in the exposed males whereas the unexposed ones (not shown here) showed only a single expected band. The additional bands were detected in several other STSs mentioned in the text.
(0.40 MB PDF)

Figure S2 Validation of Real Time PCR primers and TaqMan Probes for the DAZ genes. The assay was designed on the 100 bp fragment of sY587. (A) Real Time PCR plot for primers checked with SYBR green dye and 10 fold dilution series of human genomic DNA and recombinant plasmid for sY587. Note the Ct difference of ∼3.3 among different dilutions suggesting maximum efficiency of the reaction. (B) Standard curve obtained on the basis of the 10 fold dilution series. Note the slope and R2 values, both of which highlight the maximum efficiency of the primers. (C) Copy number calculation of the DAZ gene in normal males (ΔCt = −1) corresponding to 4 copies. (D) Copy number calculation of the DAZ gene in sperm (haploid DNA) of normal males. Note the ΔCt = −2 corresponding to four copies (see text for details). The X-Axis is cycle number and the Y, measure of Fluorescence.
(0.42 MB PDF)

Figure S3 Real time plots for the copy number calculation of the SRY gene in the males exposed to NBR. (A) Representative plot for duplication of the SRY leading to 2 copies instead of 1 (ΔCt = 0). (B) Representative plot for two rounds of duplication of the SRY leading to 4 copies (ΔCt = −1). (C) Representative plot for three rounds of duplication of the SRY leading to 8 copies (ΔCt = −2). (D) Representative plot for 4 rounds of duplication of the SRY leading to 16 copies.
(0.49 MB PDF)

Figure S4 Real time plots for the copy number calculation of the autosomal CDYL and Y linked HSFY genes in males exposed to NBR. The color coding for the lines in the plots are given above where red is for internal control RNaseP, brown, CDYL and blue for HSFY. (A) Representative plot for single copy HSFY and two copies of the CDYL genes. (B) Representative plot for duplication of the HSFY leading to 2 copies (ΔCt = 0) whereas the CDYL remains 2 copies. (C) Representative plot for the duplication of CDYL whereas the HSFY remains unaffected.
(0.40 MB PDF)

Figure S5 Real time plots for the copy number calculation of the autosomal CDYL and Y linked CDY1 genes in males exposed to NBR. Note the Ct difference among RNAseP, CDYL and CDY in different plots. The copy number of each gene corresponding to Ct values is given on the plot. The color coding for the lines in the plots are given on top.
(0.52 MB PDF)

Figure S6 SRY amino acid sequence polymorphisms. Amino acids constituting the HMG box are in green. The changes are highlighted in red with yellow background. For the consequent phenotypes of these amino acid changes, see Harley et al., 2005 [15].
(1.07 MB PDF)

Figure S7 The CDY1 gene in NBR exposed males. (A) PCR amplification of full length CDY1 (2800 bp) gene from the NBR exposed males. (B) Restriction analysis of the CDY1 recombinant, pGEMT-easy plasmid with Ecor1 showing the insert fall of 2800 bp. (C) ClustalW alignment of two CDY1 nucleotide sequences from a male 6F. The two types of sequences were concluded on the basis of ∼20 recombinant plasmids sequenced. Note the nucleotide differences between two sequences from 6F.
(0.04 MB PDF)

Figure S8 Amino Acid changes in the CDY protein in few NBR exposed males. Note that some of the amino acid changes are consistent in all the NBR exposed males. Since most of the nucleotide changes were dual, all the amino acid changes detected by the ORF finder of NCBI were observed as “X”. All the amino acid changes are highlighted in red with a yellow background.
(0.02 MB PDF)

Table S1 Semen Analysis in the males exposed to NBR. LF: Liquefaction, CL: Color, VL: Volume, VY: Viscosity, DT: Dropping Test, TC: Total Count, AM: Actively Motile, SM: Sluggish Motile, NM: Non Motile. SAA: Same as Above. In all the representative cases, the liquefaction took place before 30 seconds. The motility has been assessed with in 30 min and 60 min of the sample collection. Note the highly variable sperm count in NBR males.
(0.03 MB PDF)

Table S2 Semen Analysis in the males exposed to NBR. The numbers given under each heading represents the percent sperm with that particular phenotype. Per HPF (/HPF) is per “high power field”
(0.02 MB PDF)

Table S3 SNV/SFV typing in males exposed to NBR. List of all the SNVs and STSs used to assess the intactness of the DAZ genes and AZFc region in males exposed to natural background radiations in addition to the routine STSs.
(0.03 MB PDF)

Table S4 Details of the BAC and Cosmid clones used as probes for FISH and PCR primers used for the authentication of the clones.
(0.02 MB PDF)
