# Supplementary figures and images for: Unique Signatures of Natural Background Radiation on Human Y Chromosomes from Kerala, India
Source: PLoS One. 2009 Feb 26;4(2):e4541. doi: 10.1371/journal.pone.0004541 (PMC2644265; doi:10.1371/journal.pone.0004541)

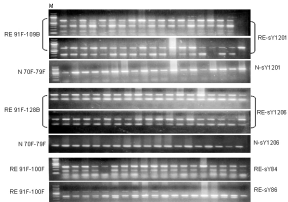

Figure S1

Supplement: Figure S1 — STSs amplifying additional bands. The ‘M’ is the molecular marker, ‘RE’ is radiation exposed, ‘N’ is normal unexposed males and, ‘F’ and ‘B’ denote father and son, respectively whereas the numbers state the respective families. Note the additional bands in case of sY1201, and sY1206 where the normal unexposed males showed only a single expected band. Similarly, in case of sY84 and sY86, multiple bands were observed in the exposed males whereas the unexposed ones (not shown here) showed only a single expected band. The additional bands were detected in several other STSs mentioned in the text. (0.40 MB PDF) [file pone.0004541.s001.pdf]

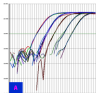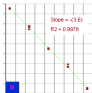

$$\Delta CT = CT_{DAZ} - CT_{RNaseP}$$

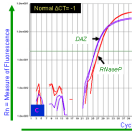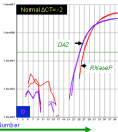

Figure S2

Supplement: Figure S2 — Validation of Real Time PCR primers and TaqMan Probes for the DAZ genes. The assay was designed on the 100 bp fragment of sY587. (A) Real Time PCR plot for primers checked with SYBR green dye and 10 fold dilution series of human genomic DNA and recombinant plasmid for sY587. Note the Ct difference of ∼3.3 among different dilutions suggesting maximum efficiency of the reaction. (B) Standard curve obtained on the basis of the 10 fold dilution series. Note the slope and R2 values, both of which highlight the maximum efficiency of the primers. (C) Copy number calculation of the DAZ gene in normal males (ΔCt = −1) corresponding to 4 copies. (D) Copy number calculation of the DAZ gene in sperm (haploid DNA) of normal males. Note the ΔCt = −2 corresponding to four copies (see text for details). The X-Axis is cycle number and the Y, measure of Fluorescence. (0.42 MB PDF) [file pone.0004541.s002.pdf]

$$\Delta CT = CT_{SRV} - CT_{RNASAP}$$

Fluorescence

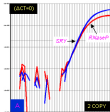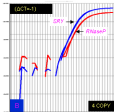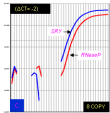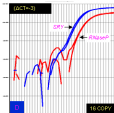

Cycle Number

Figure S3

Supplement: Figure S3 — Real time plots for the copy number calculation of the SRY gene in the males exposed to NBR. (A) Representative plot for duplication of the SRY leading to 2 copies instead of 1 (ΔCt = 0). (B) Representative plot for two rounds of duplication of the SRY leading to 4 copies (ΔCt = −1). (C) Representative plot for three rounds of duplication of the SRY leading to 8 copies (ΔCt = −2). (D) Representative plot for 4 rounds of duplication of the SRY leading to 16 copies. (0.49 MB PDF) [file pone.0004541.s003.pdf]

Rn = Measure of Fluorescence

*RNAseP*

*CDYL*

*HSFY*

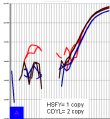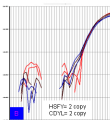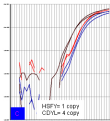

Cycle Number

Figure S4

Supplement: Figure S4 — Real time plots for the copy number calculation of the autosomal CDYL and Y linked HSFY genes in males exposed to NBR. The color coding for the lines in the plots are given above where red is for internal control RNaseP, brown, CDYL and blue for HSFY. (A) Representative plot for single copy HSFY and two copies of the CDYL genes. (B) Representative plot for duplication of the HSFY leading to 2 copies (ΔCt = 0) whereas the CDYL remains 2 copies. (C) Representative plot for the duplication of CDYL whereas the HSFY remains unaffected. (0.40 MB PDF) [file pone.0004541.s004.pdf]

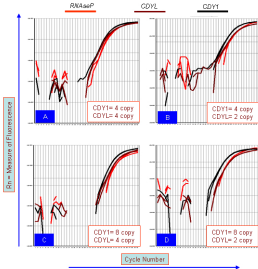

Figure S5

Supplement: Figure S5 — Real time plots for the copy number calculation of the autosomal CDYL and Y linked CDY1 genes in males exposed to NBR. Note the Ct difference among RNAseP, CDYL and CDY in different plots. The copy number of each gene corresponding to Ct values is given on the plot. The color coding for the lines in the plots are given on top. (0.52 MB PDF) [file pone.0004541.s005.pdf]
