# Supplementary material for: Unique Signatures of Natural Background Radiation on Human Y Chromosomes from Kerala, India
Source: PLoS One. 2009 Feb 26;4(2):e4541. doi: 10.1371/journal.pone.0004541 (PMC2644265; doi:10.1371/journal.pone.0004541)
Supplement: Figure S7 — The CDY1 gene in NBR exposed males. (A) PCR amplification of full length CDY1 (2800 bp) gene from the NBR exposed males. (B) Restriction analysis of the CDY1 recombinant, pGEMT-easy plasmid with Ecor1 showing the insert fall of 2800 bp. (C) ClustalW alignment of two CDY1 nucleotide sequences from a male 6F. The two types of sequences were concluded on the basis of ∼20 recombinant plasmids sequenced. Note the nucleotide differences between two sequences from 6F. (0.04 MB PDF) [file pone.0004541.s007.pdf]

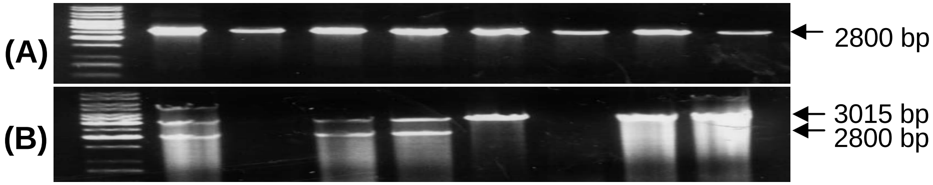

(C)

```
CDY1      TAACATTAGAATGTTCCATATAATCGTCATAGCAACCACAATTAAACAAAGAAACACAAA 600
6F_4_     ACAACCATCAAGGGCGACCCGGGCCGGNGAAAAGCTAGCTCCCGGCGGCCACGGCGGCGC 70
6f_11_    ------------------------------------------------------------

CDY1      AGAGTAACAGGCAGGAAGAAAGCTTTCTGTACTACACCAGAGGGTTGGGGCTGTGGATTT 660
6F_4_     AGAGGAATACGATTAAAGCTTATCTGTACTACACCAGAGGGTATGGGGCTGTGGATATTA 130
6f_11_    ------------------------------------------------------------

CDY1      AGCTACTCTCACCTGAGGCTACTGAGCAAGTTGTCATGCACCATGAGACAAAGCCCAAGC 720
6F_4_     GCTACTCTCACCTGAGGCTACTGAGCAAGTTGTCATGCACCATGAGACAAAGCCCAAGCT 190
6f_11_    ------------------------------------------------------------

CDY1      TGTCCCACCAGGCAGTAAGTATGGAGAGGTTCAGGCACATGGCATAGCTGCTATTTCGCA 780
6F_4_     GTCCCACCAGGCAGTAAGTATGGAGAGGTTCAGGCACATGGCATAGCTGCTATTTCGCAC 250
6f_11_    ------------------------------------------------------------

CDY1      CAATTTTCACTACACCAGTGGTGACAAAATAGAAGAGGTTCATCCATACACAGAACCTGG 840
6F_4_     AATTTTCACTACACCAGTGGTGACAAAATAGAAGAGGTTCATCCATACACAGAACCTGGT 310
6f_11_    ------------------------------------------------------------

CDY1      TGAAGAGCTGGAGGCAGAAAGAAGTGTCTATGTGGAGACGCAACTGAAACAAAGGTGGCA 900
6F_4_     GAAGAGCTGGAGGCAGAAAGAAGTGTCTATGTGGAGACGCAACTGAAACAAAGGTGGCAC 370
6f_11_    ------------------------------------------------------------

CDY1      CAGCAACTGTTCCAATCCCGTGTCTTTCCTCATGGCTTCCCAGGAGTTTGAGGTTGAAGC 960
6F_4_     AGCAACTGTTCCAAATCCCGTGTCTTTCCTCATGGCTTCCCAGGAGTTTGAGGTTGAAGC 430
6f_11_    ------------------------------------------------------------

CDY1      TATTGTTGACAAAAGACAGGATAAAAATGGGAATACACAGTATTTGGTTCGGTGGAAAGG 1020
6F_4_     TATTGTTGACAAAAGACAGGATAAAAATGGGAATACACAGTATTTGGTTCGGTGGAAAGG 490
6f_11_    ----------------CAGGATAAAAATGGGAATACACAGTATTTGGTTCGGTGGAAAGG 44
                          ********************************************

CDY1      TTATGACAAACAGGATGACACTTGGGAACCAGAGCAGCACCTCATGAACTGTGAAAAATG 1080
6F_4_     TTATGACAAACAGGATGACACTTGGGAACCAGAGCAGCACCTCATGAACTGTGAAAAATG 550
6f_11_    TTATGACAAACAGGATGACACTTGGGAACCAGAGCAGCACCTCATGAACTGTGAAAAATG 104
          ************************************************************

CDY1      TGTACATGATTTTAATAGACGACAGACTGAAAAACAGAAAAAACTGACATGGACTACAAC 1140
6F_4_     TGTACGTGATTTTAATAGACGACAGACTGAAAAACAGAAAAAACTGACATGGACTACAAC 610
6f_11_    TGTACATGATTTTAATAGACGACAGACTGAAAAACAGAAAAAACTGACATGGACTACAAC 164
          ***** ******************************************************

CDY1      CAGTAGAATTTTTTCAAACAATGCCAGAAGAAGAACTTCCAGATCTACAAAAGCAAACTA 1200
6F_4_     CAGTAGAATTTTTTCAAACAATGCCAGAAGAAGAACTTCCAGATCTACAAAAGCAAACTA 670
6f_11_    CAGTAGAATTTTTTCAAACAATGCCAGAAGAAGAACTTCCAGATCTACAAAAGCAAACTA 224
          ************************************************************

CDY1      TTCTAAGAACTCTCCTAAAACGCCAGTGACTGATAAACACCACAGGTCCAAAAACCGCAA 1260
6F_4_     TTCTAAGAACTCTCCTAAAACGCCAGTGACTGATAAACACCACAGGTCCAAAAACCGCAA 730
6f_11_    TTCTAAGAACTCTCCTAAAACTCCAGTGACTGATAAACACCACAGGTCCAAAAACTGCAA 284
          ********************* ********************************* ****
```

Figure S7

Contd/-

```
CDY1      GTTATTTGCTGCCAGCAAGAACGTTAGGAGAAAGGCAGCTTCAATTCTCTCCGACACAAA 1320
6F_4_     GTTATTTGCTGCCAGCAAGAACGTTAGGAGAAAGGCAGCTTCAATTCTCTCCGACACAAA 790
6f_11_    GTTATTTGCTGCCAGCAAGAACGTTAGGAGAAAGGCAGCTTCAACTCTCTCCGACACAAA 344
          ******************************************** ***************

CDY1      GAATATGGAGATAATAAATTCAACTATTGAGACCCTTGCACCTGACAGCCCCTTTGACCA 1380
6F_4_     GAATATGGAGATAATAAATTCAACTATTGAGACCCTTGTACCTGACAGCCCCTTTGACCA 850
6f_11_    GAATATGGAGATAATAAATTCAACTATTGAGACCCTTGCACCTGACAGCCCCTTTGACCA 404
          ************************************** *********************

CDY1      CAA---AACTGTGAGTGGCTTTCAGAAACTTGAGAAACTGGACCCTATTGCAGCAGATCA 1437
6F_4_     CAA---AACTGTGAGTGGCTTTCAGAAACTTGAGAAACTGGACCCTATTGCAGCAGATCA 907
6f_11_    CAAGAAAACTGTGAGTGGCTTTCAGAAACTTGAGAAACTGGACCCTATTGCAGCAGATCA 464
          ***   ******************************************************

CDY1      GCAGGACACGGTGGTCTTCAAGGTGACAGAAGGGAAACTCCTCCGGGACCCTTTGTCACG 1497
6F_4_     GCAGGACACGGTGGTCTTCAAGGTGACAGAAGGGAAACTCCTCCGGGACCCTTTGTCACG 967
6f_11_    GCAGGACACGGTGGTCTTCAAGGTGACAGAAGGGAAACTCCTCCGGGACCCTTTGTCACA 524
          ***********************************************************

CDY1      TCCTGGTGCAGAACAGACTGGAATACAGAACAAGACTCAGATACACCCACTAATGTCGCA 15[illegible]
6F_4_     TCCTGGTGCAGAACAGACTGGAATACAGAACAAGACTCAGATACACCCACTAATGTCGCA 10[illegible]
6f_11_    TCCTGGTGCAGAACAGACTGGAATACAGAACAAGACTCAGATGCACCCACTAATGTCGCA 58[illegible]
          ****************************************** *****************

CDY1      GATGTCTGGCTCAGTTACTGCTTCTATGGCCACAGGTTCAGCTACCCGAAAGGGTATAGT 16[illegible]
6F_4_     GATGTCTGGCTCAGTTACTGCTTCTATGGCCACAGGTTCAGCTACCCGAAAGGGTATAGT 10[illegible]
6f_11_    GATGTCTGGCTCAGTTACTGCTTCTATGGCCACAGGTTCAGCTACCCGAAAGGGTATAGT 64[illegible]
          ************************************************************

CDY1      GGTATTAATAGACCCATTAGCAGCCAATGGGACAACAGACATGCATACCTCAGTTCCAAG 16[illegible]
6F_4_     GGTATTAATAGACCCATTAGCAGCCAATGGGACAACAGACATGCATACCTCAGTTCCAAG 11[illegible]
6f_11_    GGTATTAATAGACCCATTAGCAGCCAATGGGACAACAGACATGCATACCTCAGTTCCAAG 70[illegible]
          ************************************************************

CDY1      AGTGAAAGGTGGGCAAAGAAATATTACTGATGACAGCAGAGACCAGCCTTTTATCAAGAA 17[illegible]
6F_4_     AGTGAAAGGTGGGCAAAGAAATATTACTGATGACAGCAGAGACCAGCCTTTTATCAAGAA 12[illegible]
6f_11_    AGTGAAAGGTGGGCAAAGAAATATTACTGATGACAGCAGAGGCCAGCCTTTTATCAAGAA 76[illegible]
          ***************************************** ******************

CDY1      GATGCACTTCACCATAAGGCTAACAGAAAGTGCCAGCACATACAGAGACATTGTAGTGAA 1797
6F_4_     GATGCACTTCACCATAAGGCTAACAGAAAGTGCCAGCACATACAGAGACATTGTAGTGAA 1267
6f_11_    GATGCACTTCACCATAAGGCTAACAGAAAGTGCCATCACATACAGAGACATTGTAGTGAA 824
          *********************************** ************************

CDY1      GAAAGAGGATGGATTCACCCAGATAGTGCTATCAACTAGATCGACAGAAAAAAAATGCACT 1857
6F_4_     GAAAGAGGATGGATTCACCCAGATAGTGCTATCAACTAGATCGACAGAAAAAAAATGCACT 1327
6f_11_    GAAAGAGGATGGATTCACCCAGATAGTGCTATCAACTAGATCGACAGAAAAAA-TGCACT 883
          ***************************************************** ******

CDY1      GAATACAGAAGTAATTAAAGAAATAGTTAATGCTCTGAATAGCGCTGCTGCAGATGACAG 1917
6F_4_     GAATACAGAAGTAATTAAAGAAATAGTTAATGCTCTGAATAGCGCTGCTGCAGATGACAG 1387
6f_11_    GAATACAGAAGTAATTAAAGAAATAGTTAATGCTCTGAATAGCGCTGCTGCAGATGACAG 943
          ************************************************************

CDY1      CAAGCTCGTGCTGTTCAGTGCAGCTGGAAGTGTCTTTTGCTGCGGTCTTGATTTTGGGTA 1977
6F_4_     CAAGCTCGTGCTGTTCAGTGCAGCTGGAAGTGTCTTTTGCTGCGGTCTTGATTTTGGGTA 1447
6f_11_    CAAGCTCGTGCTGTTCAGTGCAGCTGGAAGTGTCTTTTGCTGCGGTCTTGATTTTGGGTA 1003
          ************************************************************

CDY1      CTTTGTGAAGCACTTAAGGAATAACAGAAACACAGCAAGCCTTGAAATGGTGGACACCAT 2037
6F_4_     CTTTGTGAAGCACTTAAGGAATAACAGAAACACAGCAAGCCTTGAAATGGTGGACACCAT 1507
6f_11_    CTTTGTGAAGCACTTAAGGAATAACAGAAACACAGCAAGCCTTGAAATGGTGGACACCAT 1063
          ************************************************************

CDY1      CAAGAACTTTGTGAATACTTTTATTCAATTTAAAAAGCCTATTGTTGTATCAGTCAATGG 2097
6F_4_     CAAGAACTTTGTGAATACTTTTATTCAATTTAAAAAGCCTATTGTTGTATCAGTCAATGG 1567
6f_11_    CAAGAACTTTGTGAATACTTTTATTCAATTTAAAAAGCCTATTGTTGTATCAGTCAATGG 1123
          ************************************************************

CDY1      CCCTGCGATTGGACTAGGTGCATCCATCCTGCCTCTTTGTGATCTCGTGTGGGCTAATGA 2157
6F_4_     CCCTGCGATTGGACTAGGTGCATCCATCCTGCCTCTTTGTGATCTCGTGTGGGCTAATGA 1627
6f_11_    CCCTGCGATTGGACTAGGTGCATCCATCCTGCCTCTTTGTGATCTCGTGTGGGCTAATGA 1183
          ************************************************************

CDY1      AAAGGCTTGGTTCCAAACCCCTTATACGACCTTTGGACAGAGTCCAGATGGCTGTTCTTC 2217
6F_4_     AAAGGCTTGGTTCCAAACCCCTTATACGACCTTTGGACAGAGTCCAGATGGCTGTTCT-- 1685
6f_11_    AAAGGCTTGGTTCCAAACCCCTTATACGACCTTTGGACAGAGTCCAGATGGCTGTTCTTC 1243
          **********************************************************
```

Figure S7

Contd/-

```
CDY1      TATTACATTCCCCAAAATGATGGGTAAAGCATCTGCCAATGAAATGTTAATTGCTGGGCG 2277
6F_4_     ------------------------------------------------------------
6f_11_    TATTACATTCCCCAAAATGATGGGTAAAGCATCTGCCAATGAAATGTTAATTGCTGGGCG 1303

CDY1      AAAGCTGACAGCAAGGGAGGCATGCGCCAAAGGCCTGGTCTCTCAGGTATTTTTGACTGG 2337
6F_4_     ------------------------------------------------------------
6f_11_    AAAGCTGACAGCAAGGGAGGCATGCGCCAAAGGCCTGGTCTCTCAGGTATTTTTGACTGG 1363

CDY1      AACTTTCACCCAAGAGGTTATGATTCAAATTAAGGAGCTTGCCTCATACAATCCAATTGT 2397
6F_4_     ------------------------------------------------------------
6f_11_    AACTTTCACCCAAGAGGTTATGATTCAAATTAAGGAGCTTGCCTCATACAATCCAATTGT 1423

CDY1      ACTGGAAGAATGTAAGGCCCTCGTTCGCTGTAATATTAAGTTGGAGTTGGAACAGGCCAA 2457
6F_4_     ------------------------------------------------------------
6f_11_    ACTGGAAGAATGTAAGGCCCTCGTTCGCTGTAATATTAAGTTGGAGTTGGAACAGGCCAA 1483

CDY1      TGAGAGAGAGTGTGAGGTGCTGAGGAAGATCTGGAGCTCAGCCCAAGGGATAGAATCCAT 2517
6F_4_     ------------------------------------------------------------
6f_11_    TGAGAGAGAGTGTGAGGTGCTGAGGAAGATCTGGAGCTCAGCCCAAGGGATAGAATCCAT 1543

CDY1      GTTAAAGTATGTTGAAAATAAAATTGATGAGTTTTAATTGTCAGTCTGTCTGCTCAGGAC 2577
6F_4_     ------------------------------------------------------------
6f_11_    GTTAAAGTATGTTGAAAATAAAATTGATGAGTTTTAATTGTCAGTCTGTCTGCTCAGGAC 1603

CDY1      ACAAGAACTAAGGGGCAACAAATGCATCATGAGTTGCAAGATGCCCTAATCCATCTTCAT 2637
6F_4_     ------------------------------------------------------------
6f_11_    ACAAGAACTAAGGGGCAACAAATGCATCATGAGTTGCAAGATGCCCTAATCCATCTTCAT 1663

CDY1      AGCCCAAAACAATTTCACCCATAGCTAAGGCTTGGAAACAGAACTGGAAATGTCCAAGCT 2697
6F_4_     ------------------------------------------------------------
6f_11_    AGCCCAAAACAATTTCACCCATAGCTAAGGCTTGGAAACAGAACTGGAAATGTCCAAGCT 1723

CDY1      ATGTATTTAAATTATCACATCATTTTTAAGCACTGTAGCTTTACAAGGAGTAACAAAACA 2757
6F_4_     ------------------------------------------------------------
6f_11_    ATGTATTTAAATTATCACATCATTTTTAAGCACTGTAGCTTTACAAGGAGTAACAAAACA 1783

CDY1      GCCTCTTTGCCCAAATGTGATTATTTTATGCACACCTAAGCCCAAATATAAAAACAGACT 2817
6F_4_     ------------------------------------------------------------
6f_11_    GCCTCTTTGCCCAAATGTGATTATTTTATGCACACCTAAGCCCAAATATAAAAACAGACT 1843

CDY1      CTTCTTGCAAGCTCTAATATGTATCTATGGCTACTACTATATATAAGACCAGAGTTGTGT 2877
6F_4_     ------------------------------------------------------------
6f_11_    CTTCTTGCAAGCTCTAATATGTATCTATGGCTACTACTATATATAAGACCAGAGTCGTGT 1903

CDY1      TTTATTAGATGTTTGTGACAGAGAATCCTGTAATAATGTTGATTTTTTCTTATTTTTATA 2937
6F_4_     ------------------------------------------------------------
6f_11_    TTTATTAGATGTTTGTGACAGAGAATCCTGTAATAATGTTGATTTTTTCTTATTTTTATA 1963

CDY1      TCCTAGAATACCTCTGTTGGGATATAAAGCAGCCTTCCCTCCCAGAAAGACACAGAATGA 2997
6F_4_     ------------------------------------------------------------
6f_11_    TCCTAGAATACCTCTGTTGGGATATAAAGCAGCCTTCCCTCCCAGAAAGACACAGAATGA 2023

CDY1      TCAGAGATGGTGCCCTTGACTTTATAGTGGCACAAACGCTTCAGAGACACACAATTATAA 3057
6F_4_     ------------------------------------------------------------
6f_11_    TCAGAGATGGTGCCCTTGACTTTATAGTGGCACAAACGCTTCAGAGACACACAATTATAA 2083

CDY1      GAGACTTATCTTTTAGCATAAATACTTATGGCTCAAAATCCACTGACGATCATTCTCCTA 3117
6F_4_     ------------------------------------------------------------
6f_11_    GAGACTTATCTTTTAGCATAAATACTTATGGCTCAAAATCCACTGACGATCATTCTCCTA 2143

CDY1      AACTGAACACATGACTAGAATTGGTGGTGAGATATCGCTTGATTTTCTTTTCCTTTATAA 3177
6F_4_     ------------------------------------------------------------
6f_11_    AACTGAACACATGACTAGAATTGGTGGTGAGATATCGCTTGATTTTCTTTTCCTTTATAA 2203
```

Figure S7
