# Supplementary material for: Unique Signatures of Natural Background Radiation on Human Y Chromosomes from Kerala, India
Source: PLoS One. 2009 Feb 26;4(2):e4541. doi: 10.1371/journal.pone.0004541 (PMC2644265; doi:10.1371/journal.pone.0004541)
Supplement: Figure S8 — Amino Acid changes in the CDY protein in few NBR exposed males. Note that some of the amino acid changes are consistent in all the NBR exposed males. Since most of the nucleotide changes were dual, all the amino acid changes detected by the ORF finder of NCBI were observed as “X”. All the amino acid changes are highlighted in red with a yellow background. (0.02 MB PDF) [file pone.0004541.s008.pdf]

```
CDY1      -------------------MASQEFEVEAIVDKRQDKNGNTQYLVRWKGYDKQDDTWEPE 41
70F       -------------------MASQEFEVEAIVDKRQDKNGNTQYLVRWKGYDKQDDTWEPE 41
71F       -------------------MASQEFEVEAIVDKRQDKNGNTQYLVRWKGYDKQDDTWEPE 41
72F       -------------------MASQEFEVEAIVDKRQDKNGNTQYLVRWKGYDKQDDTWEPE 41
73F       -------------------MASQEFEVEAIVDKRQDKNGNTQYLVRWKGYDKQDDTWEPE 41
74F       -------------------MASQEFEVEAIVDKRQDKNGNTQYLVRWKGYDKQDDTWEPE 41
75F       ETQLKQRWHSNCSNPVSFLMASQEFEVEAIVDKRQDKNGNTQYLVRWKGYDKQDDTWEPE 120
76F       ----------------------------------QDKNGNTQYLVRWKGYDKQDDTWEPE 26
77F       -------------------MASQEFEVEAIVDKRQDKNGNTQYLVRWKGYDKQDDTWEPE 41
78F       -------------------MASQEFEVEAIVDKRQDKNGNTQYLVRWKGYDKQDDTWEPE 41
79F       -------------------MASQEFEVEAIVDKRQDKNGNTQYLVRWKGYDKQDDTWEPE 41
80F       -------------------MASQEFEVEAIVDKRQDKNGNTQYLVRWKGYDKQDDTWEPE 41
81F       -------------------MASQEFEVEAIVDKRQDKNGNTQYLVRWKGYDKQDDTWEPE 41
82F       -------------------MASQEFEVEAIVDKRQDKNGNTQYLVRWKGYDKQDDTWEPE 41
83F       -------------------MASQEFEVEAIVDKRQDKNGNTQYLVRWKGYDKQDDTWEPE 41
84F       -------------------MASQEFEVEAIVDKRQDKNGNTQYLVRWKGYDKQDDTWEPE 41
                                            **************************

CDY1      QHLMNCEKCVHDFNRRQTEKQKKLTWTTTSRIFSNNARRRTSRSTKANYSKNSPKTPVTD 101
70F       QHLMNCEKCVHDFNRRQTEKQKKLTWTTTSRIFSNNARRRTSRSTKANYSKNSPKTPVTD 101
71F       QHLMNCEKCVHDFNRRQTEKQKKLTWTTTSRIFSNNARRRTSRSTKANYSKNSPKTPVTD 101
72F       QHLMNCEKCVHDFNRRQTEKQKKLTWTTTSRIFSNNARRRTSRSTKANYSKNSPKTPVTD 101
73F       QHLMNCEKCVHDFNRRQTEKQKKLTWTTTSRIFSNNARRRTSRSTKANYSKNSPKTPVTD 101
74F       QHLMNCEKCVHDFNRRQTEKQKKLTWXTTSRIXXNNARRRTSRSTKANYSKNSPKTPVTD 101
75F       QHLMNCEKCVHDFNRRQTEKQKKLTWTTTSRIFSNNARRRTSRSTKANYSKNSPKTPVTD 180
76F       QHLMNCEKCVHDFNRRQTEKQKKLTWTTTSRIFSNNARRRTSRSTKANYSKNSPKTPVTD 86
77F       QHLMNCEKCVHDFNRRQTEKQKKLTWTTTSRIFSNNARRRTSRSTKANYSKNSPKTPVTD 101
78F       QHLMNCEKCVHDFNRRQTEKQKKLTWTTTSRIFSNNARRRTSRSTKANYSKNSPKTPVTD 101
79F       QHLMNCEKCVHDFNRRQTEKQKKLTWTTTSRIFSNNARRRTSRSTKANYSKNSPKTPVTD 101
80F       QHLMNCEKCVXDFXRRQTEKQKKLTWTTTSRIFSNNARRRTSRSTKANYSKNSPKTPVTD 101
81F       QHLMNCEKCVHDFNRRQTEKQKKLTWTTTSRIFSNNARRRTSRSTKANYSKNSPKTPVTD 101
82F       QHLMNCEKCVHDFNRRQTEKQKKLTWTTTSRIFSNNARRRTSRSTKANYSKNSPKTPVTD 101
83F       QHLMNCEKCVHDFNRRQTEKQKKLTWTTTSRIFSNNARRRTSRSTKANYSKNSPKTPVTD 101
84F       QHLMNCEKCVHDFNRRQTEKQKKLTWTTTSRIFSNNARRRTSRSTKANYSKNSPKTPVTD 101
          ********** ** ************ *****  **************************

CDY1      KHHRSKNRKLFAASKNVRRKAASILSDTKNMEIINSTIETLAPDSPFDHKTVSGFQKLEK 161
70F       KHHRSKNRKLFAASKNVRRKAASXLSDTKNMEIINSTIETLAPDSPFDHKTVSGFQKLEK 161
71F       KHHRSKNRKLFAASKNVRRKAASXLSDTKNMEIINSTIETLAPXXPXXXKXVSGFQKLEK 161
72F       KHHRSXNRKLFAAXKNVRRKAASILSDTKNMEIINSTIETLAPDSPXXHKXXSGFQKLEK 161
73F       KHHRSKNXKLFAASKNVRRKAASXLSDTKNMEIINSTIETLAPDXPXDHKXXSGFQKLEK 161
74F       KHHRSKNXKLFAASKNVRRKAASXLSDTKNMEIINSTIETLAPDXPXXXKTVSGFQKLEK 161
75F       KHHRSKNRKLFAASKNVRRKAASXLSDTKNMEIINSTIETLAPXXPXXHKXVSGFQKLEK 240
76F       KHHRSKNXKLFAASKNVRRKAASILSDTKNMEIINSTIETLAPXXPXXXKXXXGFQKLEK 146
77F       KHHRSKNXKLFAASKNVRRKAASXLSDTKNMEIINSTIETLAPXXPXXHKXXXGFQKLEK 161
78F       KHHRSKNXKLFAASKNVRRKAASXLSDTKNMEIINSTIETLAPXXPXXXKXVSGFQKLEK 161
79F       KHHRSXNXKLFAASKNVRRKAASXLSDTKNMEIINSTIETLAXXXPXXHKXVSGFQKLEK 161
80F       KHHRSKNRKLFAASKNVRRKAASXLSDTKNMEIINSTIETLAPDXPXXHKXVSGFQKLEK 161
81F       KHHRSKNXKLFAASKNVRRKAASXLSDTKNMEIINSTIETLAPDSPXXHKXXSGFQKLEK 161
82F       KHHRSKNRKLFAASKNVRRKAASXLSDTKNMEIINSTIETLAPDXPXXXKXXXGFQKLEK 161
83F       KHHRSKNXKLFAASKNVRRKAASXLSDTKNMEIINSTIETLAPDSPXXXKXXXGFQKLEK 161
84F       KHHRSKNXKLFAASKNVRRKAASXLSDTKNMEIINSTIETLAPDXPXXXKXXXGFQKLEK 161
          ***** * ***** ********* ******************   *   *   *******

CDY1      LDPIAADQQDTVVFKVTEGKLLRDPLSRPGAEQTGIQNKTQIHPLMSQMSGSVTASMATG 221
70F       LDPIAADQQDTVVFKVTEGKLLRDPLSXPGAEQTGIQNKTQXHPLMSQMSGSVTASMATG 221
71F       LDPIAADQQDTVVFKVTEGKLLRDPLSXPGAEQTGIQNKTQXHPLMSQMSGSVTASMATG 221
72F       LDPIAADQQDTVVFKVTEGKLLRDPLSXPGAEQTGIQNKTQXHPLMSQXSGSVTASMATG 221
73F       LDPIAADQQDTVVFKVTEGKLLRDPLSXPGAEQTGIQNXTQIHPLMSQMSGSVTASMATG 221
74F       LDPIAADQQDTVVFKVTEGKLLRDPLSXPGAEQTGIQNKTQXHPLMSQMSGSVTASMATG 221
75F       LDPIAADQQDTVVFKVTEGKLLRDPLSXPGAEQTGIQNKTQXHPLMSQMSGSVTASMATG 300
76F       LDPIAADQQDTVVFKVTEGKLLRDPLSXPGAEQTGIQNKTQIHPLMSQMSGSVTASMATG 206
77F       LDPIAADQQDTVVFKVTEGKLLRDPLSXPGAEQTGIQNKTQXHPLMSQMSGSVTASMATG 221
78F       LDPIAADQQDTVVFKVTEGKLLRDPLSXPGAEQTGIQNKTQXHPLMSQMSGSVTASMATG 221
79F       LDPIAADQQDTVVFKVTEGKLLRDPLSXPGAEQTGIQNKTQXHPLMSQMSGSVTASMATG 221
80F       LDPIAADQQDTVVFKVTEGKLLRDPLSXPGAEQTGIQNKTQXHPLMSQMSGSVTASMATG 221
81F       LDPIAADQQDTVVFKVTEGKLLRDPLSXPGAEQTGIQNKTQXHPLMSQMSGSVTASMATG 221
82F       LDPIAADQQDTVVFKVTEGKLLRDPLSXPGAEQTGIQNKTQXHPLMSQMSGSVTASMATG 221
83F       LDPIAADQQDTVVFKVTEGKLLRDPLSXPGAEQTGIQNKTQXHPLMSQMSGSVTASMATG 221
84F       LDPIAADQQDTVVFKVTEGKLLRDPLSXPGAEQTGIQNXTQXHPLMSQMSGSVTASMATG 221
          *************************** ********** ** ****** ***********

CDY1      SATRKGIVVLIDPLAANGTTDMHTSVPRVKGGQRNITDDSRDQPFIKKMHFTIRLTESAS 281
70F       SATRKGIVVLIDPLAANGTTDMHTSVPRVKGGQRNITDDSRXQPFIKKMHFTIRLTESAS 281
71F       SATRKGIVVLIDPLAANGTTDMHTSVPRVKGGQRNITDDSRDQPFIKKMHFTIRLTESAS 281
72F       SATRKGIVVLIDPLAANGTTDMHTSVPRVKGGQRNITDDSRXQPFIKKMHFTIRLTESAX 281
73F       SATRKGIVVLIDPLAANGTTDMHTSVPRVKGGQRNITDDSRXQPFIKKMHFTIRLTESAX 281
74F       SATRKGIVVLIDPLAANGTTDMHTSVPRVKGGQRNITDDSRXQPFIKKMHFTIRLTESAX 281
75F       SATRKGIVVLIDPLAANGTTDMHTSVPRVKGGQRNITDDSRXQPFIKKMHFTIRLTESAX 360
76F       SATRKGIVVLIDPLAANGTTDMHTSVPRVKGGQRNITDDSRDQPFIKKMHFTIRLTESAX 266
77F       SATRKGIVVXIXPLAANGTTDMHTSVPRVKGGQRNITDDSRDQPFIKKMHFTIRLTESAS 281
78F       SATRKGIVVLIDPXAANGTTDMHTSVPRVKGGQRNITDDSRXQPFIKKMHFTIRLTESAS 281
79F       SATRKGIVVLIDPLAANGTTDMHTSVPRVKGGQRNITDDSRDQPFIKKMHFTIRLTESAS 281
80F       SATRKGIVVLIDPLAANGTTDMHTSVPRVKGGQRNITDDSRDQPFIKKMHFTIRLTESAX 281
81F       SATRKGIVVLIDPLAANGTTDMHTSVPRVKGGQRNITDDSRXQPFIKKMHFTIRLTESAX 281
82F       SATRKGIVVLIDPLAANGTTDMHTSVPRVKGGQRNITDDSRXQPFIKKMHFTIRLTESAX 281
83F       SATRKGIVVLIDPLAANGTTDMHTSVPRVKGGQRNITDDSRXQPFIKKMHFTIRLTESAX 281
84F       SATRKGIVVLIDPLAANGTTDMHTSVPRVKGGQRNITDDSRDQPFIKKMHFTIRLTESAX 281
          ********* * * *************************** *****************

CDY1      TYRDIVVKKEDGFTQIVLSTRSTEKNALNTEVIKEIVNALNSAAADDSKLVLFSAAGSVF 341
70F       TYRDIVVKKEDGFTQIVLSTRSTEKNALNTEVIKEXVNALNSAAADDSKLVLFSAAGSVF 341
71F       TYRDIVVKKEDGFTQIVLSTRSTEKNALNTEVIKEIVNALNSAAADDSKLVLFSAAGSVF 341
72F       TYRDIVVKKEDGFTQIVLSTRSTEKNALNTEVIKEXVNALNSAAADDSKLVLFSAAGSVF 341
73F       TYRDIVVKKEDGFTQIVLSTRSTEKNALNTEVIKEXVNALNSAAADDSKLVLFSAAGSVF 341
74F       TYRDIVVKKEDGFTQIVLSTRSTEKNALNTEVIKEXVNALNSAAADDSKLVLFSAAGSVF 341
75F       TYRDIVVKKEDGFTQIVLSTRSTEKNALNTEVIKEXVNALNSAAADDSKLVLFSAAGSVF 420
76F       TYRDIVVKKEDGFTQIVLSTRSTEKNALNTEVIKEIVNALNSAAADDSKLVLFSAXGSVF 326
77F       TYRDIVVKKEDGFTQIVLSTRSTEKNALNTEVIKEIVNALNSAAADDSKLVLFSAAGSVF 341
78F       TYRDIVVKKEDGFTQIVLSTRSTEKNALNTEVIKEXVNALNSAAADDSKLVLFSAAGSVF 341
79F       TYRDIVVKKEDGFTQIVLSTRSTEKNALNTEVIKEIVNALNSAAADDSKLVLFSAAGSVF 341
80F       TYRDIVVKKEDGFTQIVLSTRSTEKNALNTEVIKEIVNALNSAAADDSKLVLFSAAGSVF 341
81F       TYRDIVVKKEDGFTQIVLSTRSTEKNALNTEVIKEXVNALNSAAADDSKLVLFSAAGSVF 341
82F       TYRDIVVKKEDGFTQIVLSTRSTEKNALNTEVIKEXVNALNSAAADDSKLVLFSAAGSVF 341
83F       TYRDIVVKKEDGFTQIVLSTRSTEKNALNTEVIKEXVNALNSAAADDSKLVLFSAAGSVF 341
84F       TYRDIVVKKEDGFTQIVLSTRSTEKNALNTEVIKEXVNALNSAAADDSKLVLFSAAGSVF 341
          *********************************** ******************* ****
```

Figure S8

Contd/-

```
CDY1      CCGLDFGYFVKHLRNNRNTASLEMVDTIKNFVNTFIQFKKPIVVSVNGPAIGLGASILPL 401
70F       CCGLDFGYFVXHLRNXRNTASLEMVDTIKNFVNTFIQFKKPIVVSVNGPAIGLGASILPL 401
71F       CCGLDFGYFVXHLRNXRNTASLEMVDTIKNFVNTFIQFKKPIVVSVNGPAIGLGASILPL 401
72F       CCGLDFGYFVXHLRNXRNTASLEMVDTIKNFVNTFIQFKKPIVVSVNXPAIGLGASILPL 401
73F       CCGLDFGYFVXHLRNXRNTASLEMVDTIKNFVNTFIQFKKPIVVSVNGPAIGLGASILPL 401
74F       CCGLDFGYFVXHLRNXRNTASLEMVDTIKNFVNTFIQFKKPIVVSVNGPAIGLGASILPL 401
75F       CCGLDFGYFVXHLRNXRNTASLEMVDTIKNFVNTFIQFKKPIVVSVNGPAIGLGASILPL 480
76F       CCGLDFGYFVXHLRNXRNTASLEMVDTIKNFVNTFIQFKKPIVVSVNGPAIGLGASILPL 386
77F       CXGLDFGYFVXHLRNXRNTASLEMVDTIKNFVNTFIQFKKPIVVSVNGPAIGLGASILPL 401
78F       CCGLDFGYFVXHLRNXRNTASLEMVDTIKNFVNTFIQFKKPIVVSVNGPAIGLGASILPL 401
79F       CCGLDFGYFVXHLRNXRNTASLEMVDTIKNFVNTFIQFKKPIVVSVNGPAIGLGASILPL 401
80F       CCGLDFGYFVKHLRNXRNTASLEMVDTIKNFVXTXIQFKKPIVVSVNGPAIGLGASILPL 401
81F       CCGLDFGYFVXHLRNXRNTASLEMVDTIKNFVNTFIQFKKPIVVSVNGPAIGLGASILPL 401
82F       CCGLDFGYFVXHLRNXRNTASLEMVDTIKNFVXTFIQFKKPIVVSVNGPAIGLGASILPL 401
83F       CCGLDFGYFVXHLRNXRNTASLEMVDTIKNFVNTFIQFKKPIVVSVNGPAIGLGASILPL 401
84F       CCGLDFGYFVXHLRNXRNTASLEMVDTIKNFVNTFIQFKKPIVVSVNGPAIGLGASILPL 401
          * ******** **** **************** * ************ ************

CDY1      CDLVWANEKAWFQTPYTTFGQSPDGCSSITFPKMMGKASANEMLIAGRKLTAREACAKGL 461
70F       CDLVWANEKAWFQTPYTTFGQSPDGCSSITFPKMMGKASANEMLIAGRKLTAXEACAKGL 461
71F       CDLVWANEKAWFQTPYTTFGQSPDGCSSITFPKMMGKASANEMLIAGRKLTAREACAKGL 461
72F       CDLVWANEKAWFQTPYTTFGQSPDGCSSITFPKMMGKASANEMLIAGRKLTAREACAKGL 461
73F       CDLVWANEKAWFQTPYTTFGQSPDGCSSITFPKMMGKASANEMLIAGRKLTAREACAKGL 461
74F       CDLVWANEKAWFQTPYTTFGQSPDGCSSITFPKMMGKASANEMLIAGRKLTAREACAKGL 461
75F       CDLVWANEKAWFQTPYTTFGQSPDGCSSITFPKMMGKASANEMLIAGRKLTAREACAKGL 540
76F       CDLVWANEKAWFQTPYTTFGQSPDGCSSITFPKMMGKASANEMLIAGRKLTAREACAKGL 446
77F       CDLVWANEKAWFQTPYTTFGQSPDGCSSITFPKMMGKASANEMLIAGRKLTAREACAKGL 461
78F       CDLVWANEKAWFQTPYTTFGQSPDGCSSITFPKMMGKASANEMLIAGRKLTAREACAKGL 461
79F       CDLVWANEKAWFQTPYTTFGQSPDGCSSITFPKMMGKASANEMLIAGRKLTAREACAKGL 461
80F       CDLVWANEKAWFQTPYTTFGQSPDGCSSITFPKMMGKASANEMLIAGRKLTAREACAKGL 461
81F       CDLVWANEKAWFQTPYTTFGQSPDGCSSITFPKMMGKASANEMLIAGRKLTAREACAKGL 461
82F       CDLVWANEKAWFQTPYTTFGQSPDGCSSITFPKMMGKASANEMLIAGRKLTAREACAKGL 461
83F       CDLVWANEKAWFQTPYTTFGQSPDGCSSITFPKMMGKASANEMLIAGRKLTAREACAKGL 461
84F       CDLVWANEKAWFQTPYTTFGQSPDGCSSITFPKMMGKASANEMLIAGRKLTAREACAKGL 461
          **************************************************** *******

CDY1      VSQVFLTGTFTQEVMIQIKELASYNPIVLEECKALVRCNIKLELEQANERECEVLRKIWS 521
70F       VSQVFLTGTFTQEVMIQIKELASYNXIVLEECKALVRCNIKLELEQANERECEVLRKIWS 521
71F       VSQVFLTGTFTQEVMIQXKELASYNXIVLEECKALVRCNIKLELEQANERECEVLRKIWS 521
72F       VSQVFLTGTFTQEVMIQIKELASYNXIVLEECKALVRCNIKLELEQANERECEVLRKIWS 521
73F       VSQVFLTGTFTQEVMIQIKELASYNXIVLEECKALVRCNIKLELEQANERECEVLRKIWS 521
74F       VSQVFLTGTFTQEVMIQIKELASYNXIVLEECKALVRCNIKLELEQANERECEVLRKIWS 521
75F       VSQVFLTGTFTQEVMIQIKELASYNXIVLEECKALVRCNIKLELEQANERECEVLRKIWS 600
76F       VSQVFLTGTFTQEVMIQIKELASYNXIVLEECKALVRCNIKLELEQANERECEVLRKIWS 506
77F       VSQVFLTGTFTQEVMIQIKELASYNXIVLEECKALVRCNIKLELEQANERECEVLRKIWS 521
78F       VSQVFLTGTFTQEVMIQIKELASYNXIVLEECKALVRCNIKLELEQANERECEVLRKIWS 521
79F       VSQVFLTGTFTQEVMIQIKELASYNXIVLEECKALVRCNIKLELEQANERECEVLRKIWS 521
80F       VSQVFLTGTFTQEVMIQIKELASYNXIVLEECKALVRCNIKLELEQANERECEVLRKIWS 521
81F       VSQVFLTGTFTQEVMIQIKELASYNXIVLEECKALVRCNIKLELEQANERECEVLRKIWS 521
82F       VSQVFLTGTFTQEVMIQIKELASYNXIVLEECKALVRCNIKLELEQANERECEVLRKIWS 521
83F       VSQVFLTGTFTQEVMIQIKELASYNXIVLEECKALVRCNIKLELEQANERECEVLRKIWS 521
84F       VSQVFLTGTFTQEVMIQIKELASYNXIVLEECKALVRCNIKLELEQANERECEVLRKIWS 521
          ***************** ******* **********************************

CDY1      SAQGIESMLKYVENKIDEF 540
70F       SAQGIESMLKYVENKIDEF 540
71F       SAQGIESMLKYVENKIDEF 540
72F       SAQGIESMLKYVENKIDEF 540
73F       SAQGIESMLKYVENKIDEF 540
74F       SAQGIESMLKYVENKIDEF 540
75F       SAQGIESMLKYVENKIDEF 619
76F       SAQGIESMLKYVENKIDEF 525
77F       SAQGIESMLKYVENKIDEF 540
78F       SAQGIESMLKYVENKIDEF 540
79F       SAQGIESMLKYVENKIDEF 540
80F       SAQGIESMLKYVENKIDEF 540
81F       SAQGIESMLKYVENKIDEF 540
82F       SAQGIESMLKYVENKIDEF 540
83F       SAQGIESMLKYVENKIDEF 540
84F       SAQGIESMLKYVENKIDEF 540
          *******************
```

Figure S8
