# Supplementary material for: Unique Signatures of Natural Background Radiation on Human Y Chromosomes from Kerala, India
Source: PLoS One. 2009 Feb 26;4(2):e4541. doi: 10.1371/journal.pone.0004541 (PMC2644265; doi:10.1371/journal.pone.0004541)
Supplement: Table S1 — Semen Analysis in the males exposed to NBR. LF: Liquefaction, CL: Color, VL: Volume, VY: Viscosity, DT: Dropping Test, TC: Total Count, AM: Actively Motile, SM: Sluggish Motile, NM: Non Motile. SAA: Same as Above. In all the representative cases, the liquefaction took place before 30 seconds. The motility has been assessed with in 30 min and 60 min of the sample collection. Note the highly variable sperm count in NBR males. (0.03 MB PDF) [file pone.0004541.s009.pdf]

Table S1: Semen Analysis in the males exposed to NBR#

ID	Semen Analysis							Motility					
	LF	CL	VL	pH	VY	DT	TC	AM		SM		NM	
								½ hr	1 hr	½ hr	1 hr	½ hr	1 hr
1	30 “	Grayish White	4.5 ml	7	Less	Freely	73 M/ml	30%	20%	50%	50%	20%	30%
2	30 “	SAA	6.5 ml	7	Normal	SAA	21 M/ml	10%	Nil	60%	60%	30%	40%
3	30 “	SAA	4 ml	7.5	Normal	SAA	30 M/ml	1-2 Sluggish and non motile sperms seen/HPF					
4	30 “	SAA	5 ml	8	Less	SAA	12 M/ml	05%	Nil	45%	50%	50%	50%
5	30 “	SAA	1.5 ml	7	Less	SAA	41 M/ml	20%	10%	50%	50%	30%	40%
6	30 “	SAA	1.5 ml	7	Normal	SAA	109 M/ml	40%	30%	50%	50%	10%	20%
7	30 “	SAA	0.5 ml	7.5	Slightly Viscous	SAA	147 M/ml	20%	10%	60%	60%	20%	30%
8	30 “	SAA	3 ml	7.5	Normal	SAA	18 M/ml	05%	Nil	45%	50%	50%	60%
9	30 “	SAA	1.5 ml	7	Slightly Viscous	String From	37 M/ml	10%	05%	50%	50%	40%	45%
10	30 “	SAA	2.5 ml	8	Normal	Freely	81 M/ml	30%	20%	40%	40%	30%	40%
11	1 hr	SAA	1 ml	7	High	String Form	69 M/ml		10%		50%		40%
12	30 “	SAA	2 ml	9	Less	Freely	31 M/ml	15%	10%	60%	60%	25%	30%

LF: Liquefaction, CL: Color, VL: Volume, VY: Viscosity, DT: Dropping Test, TC: Total Count, AM: Actively Motile, SM: Sluggish Motile, NM: Non Motile. SAA: Same as Above. In all the representative cases, the liquefaction took place before 30 seconds. The motility has been assessed with in 30 min and 60 min of the sample collection. Note the highly variable sperm count.
