# Supplementary material for: Unique Signatures of Natural Background Radiation on Human Y Chromosomes from Kerala, India
Source: PLoS One. 2009 Feb 26;4(2):e4541. doi: 10.1371/journal.pone.0004541 (PMC2644265; doi:10.1371/journal.pone.0004541)
Supplement: Table S2 — Semen Analysis in the males exposed to NBR. The numbers given under each heading represents the percent sperm with that particular phenotype. Per HPF (/HPF) is per “high power field” (0.02 MB PDF) [file pone.0004541.s010.pdf]

Table S2: Morphology of the Sperms in males exposed to NBR#

ID	Sperm Morphology								
	**Normal Forms**	**Round Head**	**Pin Head**	**Double Head**	**Giant Head**	**Swollen Neck**	**Curly Tail**	**Pus Cells/HPF**	**RBC's/ HPF**
1	80	02	05	01	03	04	05	2-4	1-2
2	80	02	04	02	04	04	04	8-10	2-4
3	-	-	-	-	-	-	-	4-6	2-3
4	80	06	03	02	04	02	03	8-12	
5	80	06	02	03	05	92	92	4-6	1-2
6	80	05	04	03	02	04	02	2-3	0-1
7	80	05	04	02	04	02	03	2-3	0-1
8	80	05	04	03	02	03	03	2-4	1-2
9	80	05	04	02	02	03	04	8-10	4-6
10	80	04	04	01	03	03	05	6-8	1-2
11	80	04	04	02	02	03	05	1-2	0-1
12	80	04	04	02	02	03	05	3-5	1-2

The numbers given under each heading represents the percent sperm with that particular phenotype. Per HPF (/HPF) is per "high power field"
