# Supplementary material for: Unique Signatures of Natural Background Radiation on Human Y Chromosomes from Kerala, India
Source: PLoS One. 2009 Feb 26;4(2):e4541. doi: 10.1371/journal.pone.0004541 (PMC2644265; doi:10.1371/journal.pone.0004541)
Supplement: Table S3 — SNV/SFV typing in males exposed to NBR. List of all the SNVs and STSs used to assess the intactness of the DAZ genes and AZFc region in males exposed to natural background radiations in addition to the routine STSs. (0.03 MB PDF) [file pone.0004541.s011.pdf]

Table S3: List of all the SNVs and STSs used to assess the intactness of the DAZ genes and AZFc region in males exposed to natural background radiations in addition to the routine STSs

DAZ-SNVs with restriction enzymes and the fragment size profiles for each DAZ allele

SNV	DAZ Region	GenBank Accession	Restriction Enzyme	DAZ copy	SNV allele	Restricted Fragments (bp)
I	5' UTR-exon1	G73167	*Fsp*1	1,2,3<br>4	A<br>B	709<br>398+311
II	exon3-exon4	G73163	*Mbo*1	1<br>2,3,4	A<br>B	182<br>122+60
III	intron 7A/B	G63907	*Taq*1	2<br>1,3,4	A<br>B	301<br>184+117
IV	intron 7H	G73168	*Alu*1	2<br>1,3,4	A<br>B	630<br>398+262
V	intron 9	G63908	*Dra*1	3,4<br>1,2	A<br>B	195+49<br>122+73+49
VI	3' DAZ	G73169	*Afl*III	1,2,3<br>4	A<br>B	431<br>248+183

AZFc-SNVs

STS/SNV	AZFc site	GenBank Accession	Restriction enzyme	AZFc amplicon	SNV allele	Restricted fragments (bp)
AZFc-P1/I	P1.1/1.2	G73351	*Dde*1	P1.1<br>P1.2	A<br>B	467<br>284+183
GOLY/I	GOLGA2LY gene	BV012733	*Hha*1	P1.1<br>P1.2	A<br>B	531<br>289+242
BPY2/1	BPY gene	BV012732	*Ecor*V	g2<br>g3<br>g1	A<br>A<br>B	470<br><br>289+181
TTY4/I	TTY4	BV012731	*Hae*III	g3<br>g1<br>g2	A<br>B<br>B	541<br>323+218

Additional DAZ STSs

DAZ locus STS	DAZ gene region	GenBank Accession	Presence in DAZ copy	Annealing Temp (^{0}C)	PCR product (bp)
DAZ-RRM3	DAZ 1+4 Intron 6b	G73171	1+4	66	473
Y-DAZ3	3'DAZ	G73170	3	66	260
sY152	DAZ1+4 intron 6a	G12002	1+4	66	125
