# Supplementary material for: Unique Signatures of Natural Background Radiation on Human Y Chromosomes from Kerala, India
Source: PLoS One. 2009 Feb 26;4(2):e4541. doi: 10.1371/journal.pone.0004541 (PMC2644265; doi:10.1371/journal.pone.0004541)
Supplement: Table S4 — Details of the BAC and Cosmid clones used as probes for FISH and PCR primers used for the authentication of the clones. (0.02 MB PDF) [file pone.0004541.s012.pdf]

Table S4: Details of the BAC and Cosmid clones used as probes for FISH and PCR primers used for the authentication of the clones.

S.N.	Clone	GenBank	Gene/Amplicon	Authentication of the clones by PCR 5'-3'	Probe ID'S
1	M18E8	AC010089	5' *DAZ* (Exon 1-7)	Saxena R. et al. 2000 , sY579<br>A1F GCCAATTATCTCCAAATTATGGATTC (1455-1480)<br>A1R CACATAGCAGGAATACGTCTGACTTT (2469-2494)<br>A2F GGGAAAGAATTGTCTTTAATGATGCT (13229-13254)<br>A2R CAGTTACCTTTTCTGCTCCTCTCTCT(14529-554)<br>A3F AGGAATTGCTCACAACAATCAGTTAC(25138-25163)<br>A3R AGTTAGCCCATAAAAGCAAATGTAGG(26445-26470)<br>A4F CGTTCAGATTCAGGAAATACAGAGAA(41035-060)<br>A4R AGTTCTAGTTTGATTGCACTGTGGTC(42219-42244)	A
2	M63C9	AC000021	Complete *DAZ* region (Exon 2-11)	Saxena R. et al. 2000<br>sY587	B
3	46A6	AC000022	3' *DAZ* region	Saxena R. et al. 2000<br>sY586	C
4	336F2		g1/g2/g3 amplicon (AZFc)	Repping S. et al. 2003	D
