# Supplementary material for: Unique Signatures of Natural Background Radiation on Human Y Chromosomes from Kerala, India
Source: PLoS One. 2009 Feb 26;4(2):e4541. doi: 10.1371/journal.pone.0004541 (PMC2644265; doi:10.1371/journal.pone.0004541)
Supplement: Figure S6 — SRY amino acid sequence polymorphisms. Amino acids constituting the HMG box are in green. The changes are highlighted in red with yellow background. For the consequent phenotypes of these amino acid changes, see Harley et al., 2005 [15]. (1.07 MB PDF) [file pone.0004541.s006.pdf]

```
SRY    MQSYASAMLSVFNSDDYSPAVQENIPALRRSSSFLCTESCNSKYQCETGENSKGNVQDRV 60
70F    -------MLSVFNSDDYSPAVQENIPALRRSSSFLCTESCNSKYQCETGENSKGNVQDRV 53
70B    MRSYASAMLSVFNSDDYSPAVQENIPALRRSSSFLCTESCNSKYQCETGENSKGNVQDRV 60
71F    MQSYASAMLSVFNSDDYSPAVQENIPALRRSSSFLCTESCNSKYQCETGENSKGNVQDRV 60
71B    MQSYASAMLSVFNSDDYSPAVQENIPALRRSSSFLCTEAVT------------------- 41
72B    MQSYASAMLSVFNSDDYSPAVQENIPALRRSSSFLCTESCNSKYQCETGENSKGNVQDRV 60
73F    -------MLSVFNSDDYSPAVQENIPALRRSSSFLCTESCNSKYQCETGENSKGNVQDRV 53
73B    -------------------LCIENIPALRRSSSFPCTESCNSKYQCGTGENSKGNVQDRV 41
74F    -------MLSVFNSDDYSPAVQENIPALRRSSSFLCTESCNSKYQCETGENSKGNVQDRV 53
74B    MQSYASAMLSVFNSDDYSPAVQENIPALRRSSSFLCTESCNSKYQCETGENSKGNVQDRV 60
75F    -------MLSVFNSDDYSPAVQENIPALRRSSSFLCTESCNSKYQCETGENSKGNVQDRV 53
75B    -------MLSVFNSDDYSPAVQENIPALRRSSSFLCTESCNSKYQCETGENSKGNVQDRV 53
76F    -------MLSVFNSDDYSPAVQENIPALRRSSSLLCTESCNSKYQCETGENSKGNVQDRV 53
76B    MQSYASAMLSVFNSDDYSPAVQENIPALRRSSSFLCTEML-------------------- 40
77F    -------MLSVFNSDDYSPAVQENIPALRRSSSFLCTESCNSKYQCETGENSKGNVQDRV 53
77B    -------MLSVFNSDDYSPAVQENIPALRRSSSFLCTESCNSRYQCETGENSKGNVQDRV 53
78F    MQSYASAMLSVFNSDDYSPAVQENIPALRRSSSFLCTESCNSKYQCETGENSKGNVQDRV 60
78B    MQSYASAMLSVFNSDDYSPAVQENIPALRRSSSFLCTESCNSKYQCETGENSKGNVQDRV 60
79F    MQSYASAMLSVFNSDDYSPAVQENIPALRRSSSFLCTESCNSKYQCETGENSKGNAQDRV 60
79B    MQSYASAMLSVFNSDDYSSAVQEDIPALRRSSSFLCTESCNSKYQCETGENSKGNVQDRV 60
                             *:*********: ***

SRY    KRPMNAFIVWSRDQRRKMALENPRMRNSEISKQLGYQWKMLTEAEKWPFFQEAQKLQAMH 120
70F    KRPMNAFIVWSRDQRRKMALENPRMRNSEISKQLGYQWKMLTEAEKWPFFQEAQKLQAMH 113
70B    KRPMNAFIVWSRDQRRKMALENPRMRNSEISKQLGYQWKMLTEAEKWPFFQEAQKLQAMH 120
71F    KRPMNAFIVWSRDQRRKMALENPRMRNSEISKQLGYQWKMLTEAEKWPFFQEAQKLQAMH 120
71B    -------------------LS--------IS----------------------------- 45
72B    KRPMNAFIVWSRDQRRKMALENPRMRNSEISKQLGYQWKMLTEAGKWPFFQEAQKLQAMH 120
73F    KRPMNAFIVWSRDQRRKMALENPRMRNSEISKQLGYQWKMLTEAEKWPFLQEAQKLQAMH 113
73B    KRPMNAFIVWSRDQRRKMALENPRMRNSEISKQLGYQWKMLTEAEKWPFFQEAQKLQAMH 101
74F    KRPMNAFIVWSRDQRRKMALENPRMRNSEISKQLGYQWKMLTEAEKWPFFQEAQKLQAMH 113
74B    KRPMNAFIVWSRDQRRKMALENPRMRNSEISKQLGYQWKMLTEAEKWPFFQEAQKLQAMH 120
75F    KRPMNAFIVWSRDQRRKMALENPRMRNSEISKQLGYQWKMLTEAEKWPFFQEAQKLQAMH 113
75B    KRPMNAFIVWSRDQRRKMALENPRMRNSEISKQLGYQWKMLTEAEKWPFFQEAQKLQAMH 113
76F    KRPMNAFIMWSRDQRRKMALENPRMRNSEISKQLGYQWKMLTEAEKWPFFQEAQKLQAMH 113
76B    ------------------------------------------------------------
77F    KRPMNAFIVWSRDQRRKMALENPRMRNSEISKQLRYQWKMLTEAEKWPFFQEAQKLQAMH 113
77B    KRPMNAFIVWSRDQRRKMALENPRMRNSEISKQLGYQWKMLTEAEKWPFFQEAQKLQAMH 113
78F    KRPMNAFIVWSRDQRRKMALENPRMRNSEISKQLGYQWKMLTEAEKWPFFQEAQKLQAMH 120
78B    KRPMNAFIVWSRDQRRKMALENPRMRNSEISKQLGYQWKMLTEAEKWPFFQEAQKLQAMH 120
79F    KRPMNAFIVWSRDQRRKMALENPRMRNSEISKQLGYQWKMLTEAEKWPFFQEAQKLQAMH 120
79B    KRPMNAFIVWSRDQRRKMALENPRMRNSEISKQLGYQWKMLTEAEKWPFFQEAQKLQAMH 120

SRY    REKYPNYKYRPRRKAKMLPKNCSLLPADPASVLCSEVQLDNRLYRDDCTKATHSRMEHQL 180
70F    REKYPNYKYRPRRKAKMLPKNCSLLPADPASVLCSEVQLDNRLYRDDCTKATHSRMEHQL 173
70B    REKYPNYKYRPRRKAKMLPKNCSLLPADPASVLCSEVQLDNRLYRDDCTKATHSRMEHQL 180
71F    QREIPEL----------------------------------------------------- 127
71B    ------------------------------------------------------------
72F    REKYPNYKYRPRRKAKMLPKNCSLLPADPASVLCSEVQLDNRLYRDDCTKATHSRMEHQL 180
72B    REKYPNYKYRPRRKAKMLPKNCSLLPADPASVLCSEVQLDNRLYRDDCTRATHSRMEHQL 180
73F    REKYPNYKYRPRRKAKMLPKDCSLLPADPASVLCSEVQLDNRLYRDDCTKATHSRMEHQL 173
73B    REKYPNHKYRPRRKAMMLPKNCSLLPADPASVLCSEVQLDNRLHMDDCTTATHSRMEHQL 161
74F    REKYPNYKYRPRRKAKMLPKNCSLLPADPAS----------------------------- 144
74B    REKYPNYKYRPRRKAKMLPKNCSLLPADPASVLCSEVQLDNRLYRG-------------- 166
75F    REKYPNYKYRPRRKAKMLPKNCSLLPADPASVLRSEVQLDNRLHRDDCTKATHSRMEHQL 173
75B    REKYPNYKYRPRRKAKMLPKNCSLLPADPASVLCSEVQLDNRLYRDDCTKATHSRMEHQL 173
76F    REKYPNYKYRPRRKAKMLPKNCSLLPADPASVLCSEVQLDNRLYRDDCTKATHSRMEHQL 173
76B    ------------------------------------------------------------
77F    REKYPNYKYRPRRKAKMLPKNCSLLPADPASVLCSEVQLDNRLYRDDCTKATHSRMEHQL 173
77B    REKYPNYKYRPRRKAKMLPKNCSLLPADPASVLCSEVQLDNRLYRDDCTKTTHSRMEHQL 173
78F    REKYPNYKYRPRRKAKMLPKNCSLLPADPASVLCSEVQLDNRLYRDDCTKATHSRMEHQL 180
78B    REKYPNYKYRPRRKAKMLPKNCSLLPADPASVLCSEVQLDNRLYRDDCTKATHSRMEHQL 180
79B    REKYPNYKYRPRRKAKMLPKNCSLLPADPATVLCSEVQLDNRLYRG-------------- 166
79F    REKYPNYKYRPRRKAKMLPKNCSLLPADPASVLCSEVQLDNRLYRDDCTKATHSRMEHQL 180
```

**Figure S6**
